# A Longitudinal Characterization of the Seminal Microbiota and Antibiotic Resistance in Yearling Beef Bulls Subjected to Different Rates of Gain

**DOI:** 10.1128/spectrum.05180-22

**Published:** 2023-03-14

**Authors:** Emily M. Webb, Devin B. Holman, Kaycie N. Schmidt, Matthew S. Crouse, Carl R. Dahlen, Robert A. Cushman, Alexandria P. Snider, Kacie L. McCarthy, Samat Amat

**Affiliations:** a Department of Microbiological Sciences, North Dakota State University, Fargo, North Dakota, USA; b Lacombe Research and Development Centre, Agriculture and Agri-Food Canada, Lacombe, Alberta, Canada; c USDA, Agriculture Research Service, U.S. Meat Animal Research Center, Clay Center, Nebraska, USA; d Department of Animal Sciences, North Dakota State University, Fargo, North Dakota, USA; e Department of Animal Sciences, Institute of Agriculture and Natural Resources, University of Nebraska-Lincoln, Lincoln, Nebraska, USA; Texas A&M University

**Keywords:** seminal microbiota, bovine, 16S rRNA gene sequencing, culturing, rate of gain, quantitative PCR, fecal microbiota, antimicrobial resistance, core taxa, cattle

## Abstract

In this study, we evaluated the seminal and fecal microbiota in yearling beef bulls fed a common diet to achieve moderate (1.13 kg/day) or high (1.80 kg/day) rates of weight gain. Semen samples were collected on days 0 and 112 of dietary intervention (*n* = 19/group) as well as postbreeding (*n* = 6/group) using electroejaculation, and the microbiota was assessed using 16S rRNA gene sequencing, quantitative PCR (qPCR), and culturing. The fecal microbiota was also evaluated, and its similarity with seminal microbiota was assessed. A subset of seminal bacterial isolates (*n* = 33) was screened for resistance against 28 antibiotics. A complex and dynamic microbiota was detected in bovine semen, and the community structure was affected by sampling time (*R*^2^ = 0.16, *P < *0.001). Microbial richness increased significantly from day 0 to day 112, and diversity increased after breeding (*P > *0.05). Seminal microbiota remained unaffected by the differential rates of gain, and its overall composition was distinct from fecal microbiota, with only 6% of the taxa shared between them. A total of 364 isolates from 49 different genera were recovered under aerobic and anaerobic culturing. Among these seminal isolates were pathogenic species and those resistant to several antibiotics. Overall, our results suggest that bovine semen harbors a rich and complex microbiota which changes over time and during the breeding season but appears to be resilient to differential gains achieved via a common diet. Seminal microbiota is distinct from the fecal microbiota and harbors potentially pathogenic and antibiotic-resistant bacterial species.

**IMPORTANCE** Increasing evidence from human and other animal species supports the existence of a commensal microbiota in semen and that this seminal microbiota may influence not only sperm quality and fertility but also female reproduction. Seminal microbiota in bulls and its evolution and factors shaping this community, however, remain largely underexplored. In this study, we characterized the seminal microbiota of yearling beef bulls and its response to the bull age, different weight gains, and mating activity. We compared bacterial composition between seminal and fecal microbiota and evaluated the diversity of culturable seminal bacteria and their antimicrobial resistance. Our results obtained from sequencing, culturing, and antibiotic susceptibility testing provide novel information on the taxonomic composition, evolution, and factors shaping the seminal microbiota of yearling beef bulls. This information will serve as an important basis for further understanding of the seminal microbiome and its involvement in reproductive health and fertility in cattle.

## INTRODUCTION

Despite advances in genetic selection, artificial insemination, and improved cattle feeding and management over the last 4 decades, reproductive failure remains the most significant problem impacting both beef and dairy cattle production, causing serious management and economic challenges to the American cattle industry (1–3). According to a recent meta-analysis, almost 48% of beef cows experience pregnancy loss during the first 30 days of gestation following a single insemination, and approximately 6% of pregnancy loss occurs during the remaining months of gestation (3). Thus, improving fertility and reducing embryonic mortality and pregnancy loss in beef cattle are important to maintaining sustainable beef production and meeting the expected 50% increase in the demand for meat by 2050 as the global population continues to grow (4).

It is increasingly known that the reproductive tract microbiome of humans and other vertebrate animals may affect fertility, and therefore, microbiome-targeted approaches may offer novel opportunities for reducing the incidence of reproductive failures (5–7). The association of the vaginal microbiome with fertility in women has been relatively well documented (7, 8). While direct evidence is still lacking, it has been suggested that the uterine microbiome may be involved in the regulation of endometrial physiology, thereby influencing reproductive health, fertility, placentation, and healthy pregnancy (9). The perturbation of the uterine microbiota may negatively affect conception rate and increase pregnancy loss. Thus, maintaining homeostasis of the uterine microbiome is likely important in female reproductive health and fertility. One of the microbial sources seeding the uterus is believed to be semen (10, 11). The term “complementary seminovaginal microbiota” (12) has been proposed to describe the transmission of microorganisms from the male reproductive tract to the uterus (11). Several studies have demonstrated that certain bacterial species are shared between the female and male urogenital microbiota (12–14). Thus, transmission of the microorganisms from semen to the female urogenital tract could be one of the factors influencing both vaginal and uterine microbiota homeostasis.

In addition to the potential impact of microbes associated with semen on the female reproductive microbiome, emerging evidence has revealed a potential link between the seminal microbiota and sperm quality (15). For example, normal-quality human semen was shown to harbor a microbial community with a greater abundance of *Lactobacillus* and *Gardnerella* spp. than poor-quality semen (16). Likewise, the bacterial composition of semen samples was different between those with positive and negative *in vitro* fertilization (IVF) outcomes (17) and between human papillomavirus (HPV)-positive and HPV-negative semen samples (18). Similar observations in horse seminal microbiota observed, with bacterial species within the *Peptoniphilaceae* family, positively associated with stallion sperm motility (19). Together, these studies highlight a potentially significant role of the seminal microbiota in sperm development and male fertility.

There is an increased appreciation for the potential impact of the seminal microbiota on male and female fertility based on evidence from developments in human and horse observational studies, and as a result, the microbial community in bovine semen has also begun to be explored using high-throughput sequencing (20, 21). Although the presence of a microbial community in bovine semen has been reported, very little is known about the bovine seminal microbiota, its dynamics and evolution, and the factors that shape its structure and composition. The objectives of the present study were to (i) characterize the microbial community present in the semen of yearling beef bulls using 16S rRNA amplicon sequencing, (ii) evaluate whether the bovine seminal microbiota is influenced by the different rates of gain obtained via a common diet, (iii) compare seminal microbiota with fecal microbiota and identify core taxa shared between these two communities, (iv) characterize the culturable fraction of bovine seminal microbiota using aerobic and anaerobic culturing, which is also complementary to the 16S rRNA amplicon sequencing, as culturing could provide higher taxonomic resolution (at the species level) than that of amplicon sequencing, and (v) evaluate the antimicrobial resistance in bovine seminal bacterial isolates.

## RESULTS

### Bull health, weight gain, and sperm quality.

All bulls remained healthy during the 112-day feeding period. During breeding, one bull was removed from the pasture due to a hernia, and semen and fecal samples from this bull were not collected at postbreeding. As a result of dietary treatment given over the course of 112 days, the moderate and high group bulls had significantly different weight gain (moderate, 464.8 ± 4.92 kg; high, 537.6 ± 4.79 kg). As for the sperm quality, there were no differences (*P = *0.93) in the percentage of morphologically normal sperm between treatments at prebreeding or postbreeding. At prebreeding, there was no difference (*P = *0.17) in the total concentration of sperm per ejaculate or the percentage of motile sperm (Crouse et al., unpublished data).

### 16S rRNA gene sequencing overview.

An average of 81,270 ± 15,870 (standard deviation [SD]) 16S rRNA gene sequences per sample (minimum, 39,076; maximum, 116,508) were obtained from 84 seminal samples from beef bulls fed a common diet to achieve moderate or high rates of weight gain over the course of 112 days. In the 48 fecal samples, there were 72,117 ± 8,311 (SD) 16S rRNA gene sequences per sample (minimum, 52,481; maximum, 90,849). From the sequences of all seminal and fecal samples, a total of 17,194 archaeal and bacterial amplicon sequence variants (ASVs) were identified and classified into 32 unique phyla (4 archaeal and 28 bacterial phyla), 776 unique genera, and 1,184 unique species.

### Microbial community structure of the seminal microbiota.

**FIG 1:**
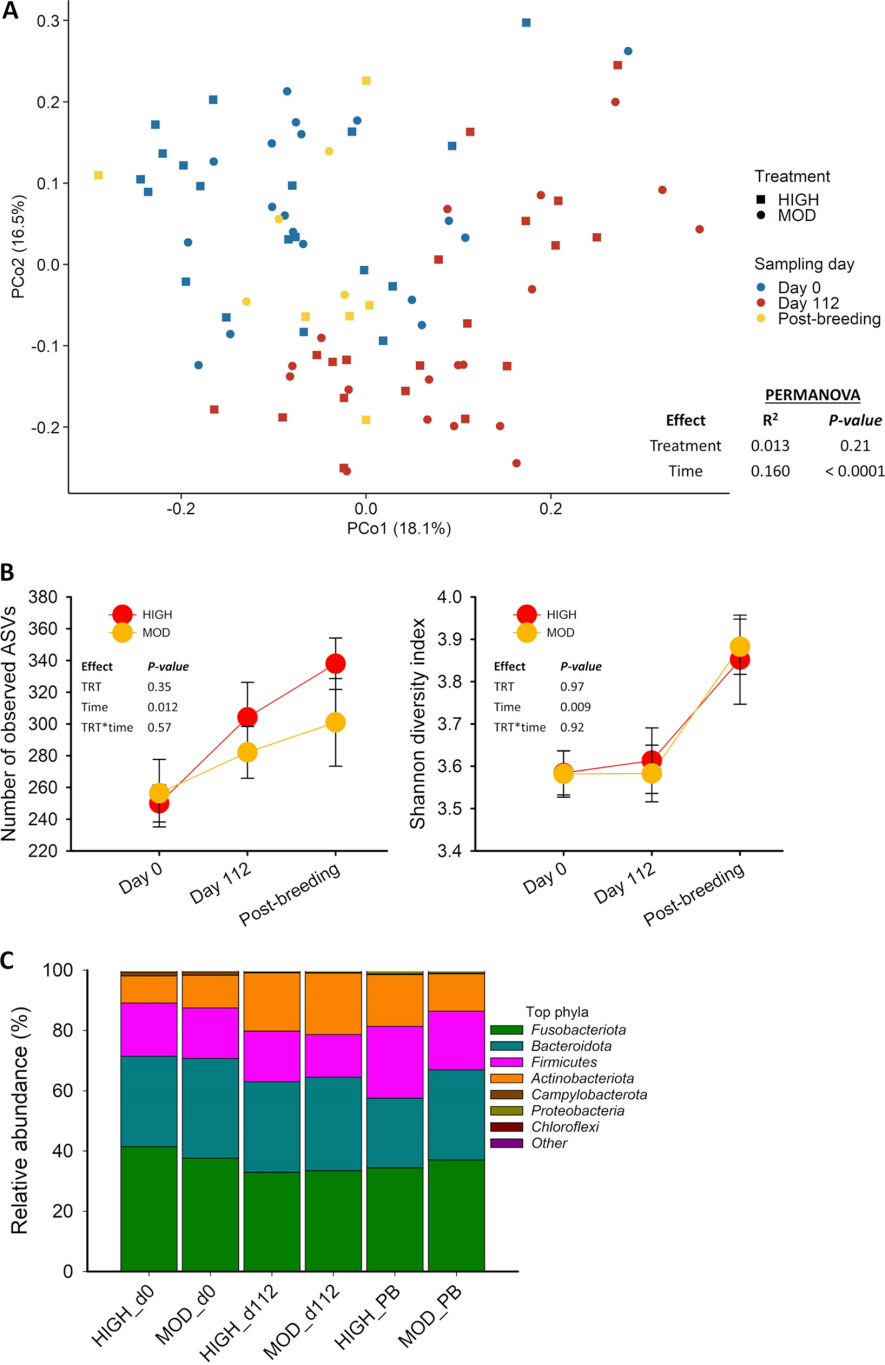
Beta and alpha diversity and microbial composition of the seminal microbiota of beef bull subjected to high (high) or moderate (moderate) rate of gains. (A) Principal-coordinate analysis (PCoA) plot of the Bray-Curtis dissimilarities. (B) Number of observed amplicon sequencing variants (ASVs) and Shannon diversity index. (C) Relative abundance of the 7 most relatively abundant bacterial phyla in the seminal microbiota.

A relatively diverse and dynamic microbial community was detected in the semen samples (Fig. 1). The community structure of the seminal microbiota underwent significant changes over the course of the study (permutational multivariate analysis of variance [PERMANOVA]; *R*^2^ = 0.16, *P < *0.001), with the greatest difference between day 0 and day 112 samples (*R*^2^ = 0.126, *P = *0.003). However, the seminal microbial community structure did not differ between the moderate and high bulls (*R*^2^ = 0.013, *P = *0.21) at any of the sampling time points. Similarly, the alpha diversity metrics of the seminal microbiota changed significantly over time (*P < *0.05) but were unaffected by dietary treatment (*P > *0.05) (Fig. 1B). Microbial richness (number of ASVs) increased from day 0 (253 ± 12) to day 112 (293 ± 14; *P < *0.05) and remained stable from day 112 to postbreeding (320 ± 23; *P > *0.05). The Shannon (Fig. 1B) and inverse Simpson diversity indices (data not shown) did not change from day 0 to day 112 but increased from day 112 to postbreeding (*P < *0.05).

Twenty-eight bacterial phyla were identified among all semen samples, with *Fusobacteriota* (36.3%), *Bacteroidota* (30.4%), *Firmicutes* (17.1%), and *Actinobacteriota* (14.9%) as the dominant phyla (Fig. 1C). Among the 7 most predominant bacterial phyla listed in Fig. 1C, the relative abundance of *Firmicutes* was lower in the moderate (16.8 ± 0.83%) than high (19.4 ± 0.72%) bulls (*P = *0.02). The relative abundance of the other dominant phyla did not change in response to dietary treatment (*P > *0.05). A significant time effect (*P < *0.05) was observed on *Firmicutes*, *Campylobacterota*, *Actinobacteriota*, and *Proteobacteria*, with most changes in abundance of these phyla occurring between day 112 and postbreeding. *Chloroflexi* and *Bacteroidota*; however, remained unaffected by time (*P ≥ *0.17).

The 32 most relatively abundant genera in seminal microbiota are listed in Table 1. *Fusobacterium* (25.9%) and *Porphyromonas* (20.4%) were the most predominant genera, accounting for more than 45% of total 16S rRNA gene sequences in the seminal microbiota. Among the most relatively abundant genera were those with beneficial and commensal members such as *Bifidobacterium*, *Prevotella*, and *Succinivibrio* as well as genera such as Campylobacter and *Trueperella* that contain potentially pathogenic species. The relative abundance of five genera (*Fastidiosipila*, *Parvimonas*, *Streptobacillus*, *Oscillospiraceae* UCG-005, and *Caviibacter*) was affected by dietary treatment (*P < *0.05) (Table 1).

**TABLE 1:** Percent relative abundance of the 32 most relatively abundant genera in the seminal microbiota of beef bulls subjected to high (high) or moderate (moderate) rate of gains by sampling time^*a*^

Genus	Rank	% abundance						SEM	*P* value		
		Day 0 at rate:		Day 112 at rate:		Postbreeding at rate:					
		High	Moderate	High	Moderate	High	Moderate		TRT	Time	TRT × time
*Fusobacterium*	1	29.68	28.18	24.81	25.39	16.41	19.67	1.160	0.58	<0.0001	0.425
*Porphyromonas*	2	21.37	22.24	20.27	19.28	14.97	21.08	1.268	0.20	0.1331	0.248
*Oceanivirga*	4	4.39	3.84	3.51	4.82	8.50	6.99	0.492	0.68	<0.0001	0.109
*Corynebacterium*	5	3.27	4.44	4.40	4.88	7.74	5.63	1.305	0.92	0.429	0.753
*Alloprevotella*	6	3.11	3.08	4.77	6.16	3.09	3.25	0.543	0.45	0.0003	0.454
*Bacteroides*	7	3.41	4.65	2.73	2.91	1.38	1.26	0.315	0.26	<0.0001	0.213
*Fastidiosipila*	8	3.16	2.25	2.95	2.56	4.82	2.30	0.391	0.01	0.408	0.271
*Mageeibacillus*	9	1.95	1.69	2.97	3.03	4.97	2.90	0.389	0.11	0.0015	0.259
*Arcanobacterium*	10	1.68	1.64	4.08	3.26	0.96	1.47	0.383	0.80	<0.0001	0.482
*Parvimonas*	12	3.38	3.06	1.69	1.32	1.70	2.53	0.261	0.88	<0.0001	0.363
*Prevotella*	13	2.04	3.00	1.54	2.12	2.03	3.29	0.339	0.03	0.116	0.783
*Leucobacter*	17	0.57	0.63	2.45	2.87	2.86	1.65	0.387	0.62	<0.0001	0.454
*Actinomyces*	18	0.83	1.05	1.33	1.73	0.58	0.41	0.160	0.45	0.0001	0.559
*[Eubacterium] brachy* group	19	1.03	1.11	0.70	0.66	0.95	1.27	0.103	0.34	0.0012	0.552
Campylobacter	20	1.26	1.21	0.19	0.19	0.25	0.27	0.105	0.94	<0.0001	0.955
*Leptotrichia*	21	0.76	0.57	0.53	0.44	0.60	1.37	0.102	0.19	0.014	0.021
*Clostridium sensu stricto* 1	22	0.55	0.63	0.44	0.35	0.99	0.48	0.143	0.32	0.255	0.462
*Helcococcus*	23	0.55	0.82	0.26	0.20	0.48	0.26	0.157	0.98	0.029	0.493
*Filifactor*	24	0.44	0.38	0.33	0.33	0.37	0.50	0.085	0.80	0.60	0.785
*Bifidobacterium*	25	0.24	0.35	0.44	0.40	0.54	0.41	0.084	0.87	0.260	0.568
*Romboutsia*	26	0.28	0.36	0.37	0.28	0.83	0.37	0.073	0.08	0.060	0.092
*Trueperella*	27	0.24	0.52	0.13	0.18	0.18	0.29	0.119	0.31	0.214	0.639
*Streptobacillus*	28	0.21	0.12	0.31	0.24	0.73	0.22	0.059	0.00	0.008	0.067
*Oscillospiraceae* UCG-005	30	0.04	0.04	0.16	0.15	0.87	0.40	0.038	0.00	<0.0001	0.001
*Peptoniphilus*	31	0.11	0.32	0.11	0.10	0.11	0.17	0.049	0.17	0.109	0.110
*Dietzia*	32	0.04	0.04	0.28	0.27	0.07	0.09	0.052	0.94	0.0002	0.978
*Succinivibrio*	33	0.00	0.00	0.13	0.17	0.69	0.62	0.054	0.87	<0.0001	0.787
*Caviibacter*	34	0.27	0.17	0.03	0.02	0.43	0.06	0.062	0.04	0.007	0.220
*Gallicola*	36	0.02	0.02	0.22	0.23	0.19	0.16	0.051	0.95	0.001	0.970
*Tessaracoccus*	37	0.06	0.10	0.14	0.12	0.10	0.08	0.026	0.98	0.167	0.530
*Howardella*	38	0.07	0.08	0.11	0.10	0.15	0.17	0.010	0.76	<0.0001	0.698
*Peptostreptococcus*	39	0.09	0.08	0.07	0.14	0.09	0.04	0.029	1.00	0.587	0.266

a The genera whose relative abundance was ranked within the top 40 are listed in this table, and any within the top 40 rank that were unclassified at the genus level were excluded. TRT, treatment.

### Microbial community composition and structure of the fecal microbiota.

**FIG 2:**
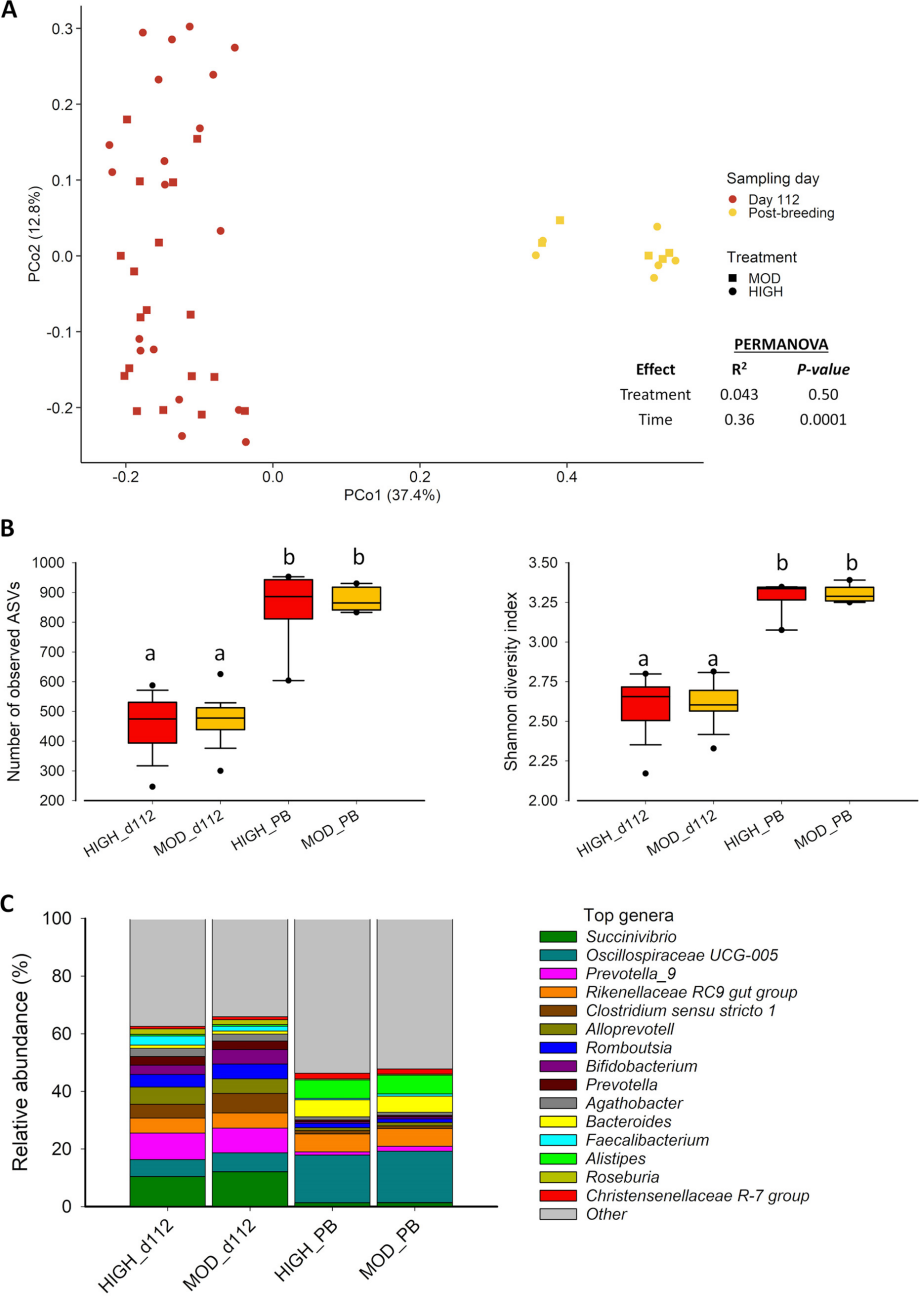
Beta and alpha diversity and microbial composition of the fecal microbiota of beef bull subjected to high (high) or moderate (moderate) rate of gains. (A) Principal-coordinate analysis (PCoA) plot of the Bray-Curtis dissimilarities. (B) Number of observed amplicon sequencing variants (ASVs) and Shannon diversity index. (C) Relative abundance of 15 most relatively abundant bacterial genera in the fecal microbiota.

The fecal microbiota structure did not differ between the moderate and high bulls at either sampling time (*R*^2^ = 0.043, *P = *0.50) but changed significantly from day 112 to postbreeding (*R*^2^ = 0.16; *P < *0.0001) (Fig. 2A). Alpha diversity metrics were affected by time but not dietary treatment (Fig. 2B). Microbial richness increased from day 112 to postbreeding, with the number of observed ASVs increasing by 47% (*P = *0.012) (Fig. 2B). Similarly, microbial diversity increased from day 112 to postbreeding (Shannon [Fig. 2B] and inverse Simpson diversity [data not shown]; *P *< 0.0001).

Microbial composition at the genus level underwent a significant change over time but remained unaffected by dietary treatment. Among the 15 most relatively abundant bacterial genera in the fecal microbiota, the relative abundance of 14 genera was significantly altered from day 112 to postbreeding (Fig. 2C). There was a sharp decline in the relative abundance of many of the dominant genera (e.g., *Succinivibrio*, *Prevotella* 9, and *Clostridium sensu stricto* 1), while few of the dominant genera, including *Oscillospiraceae* UCG-005 and *Bacteroides*, became dominant at postbreeding compared to day 112 (*P < *0.05).

### Similarity of the fecal and seminal microbiota and core taxa shared across these microbial communities.

**FIG 3:**
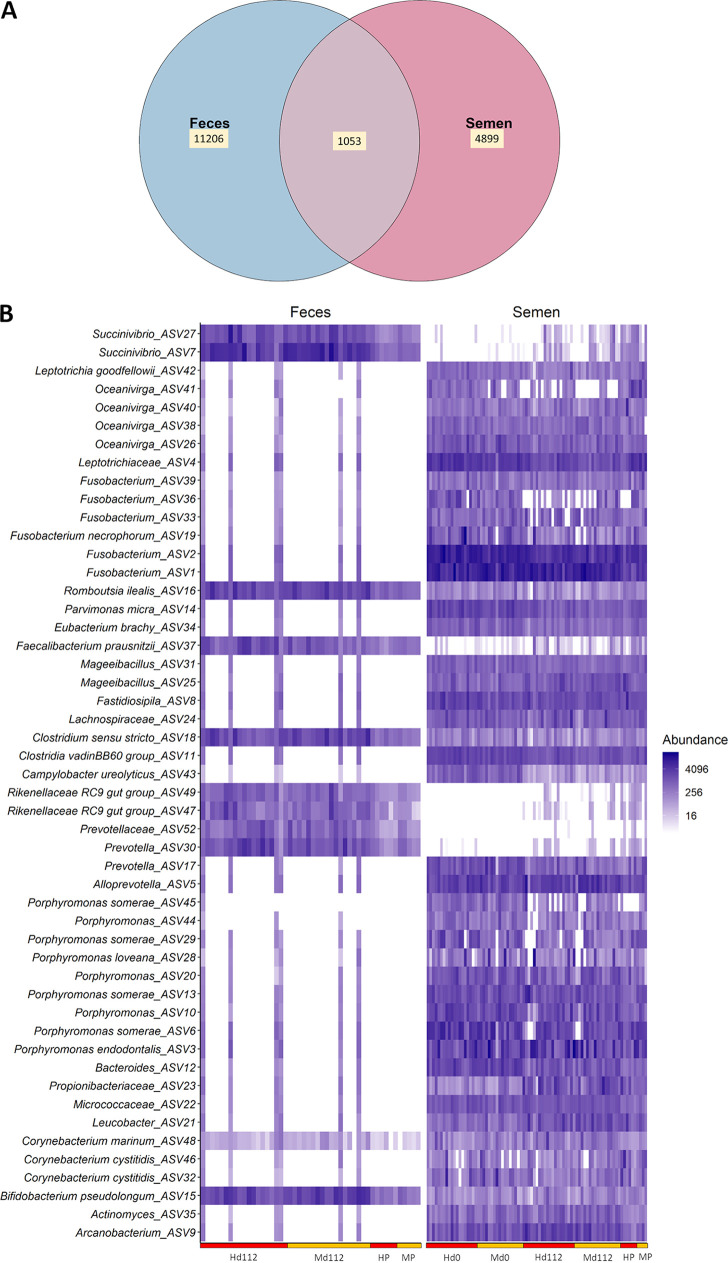
Similarity between fecal and seminal microbiota. (A) Venn diagram displaying the number of shared and unique ASVs among the seminal and fecal microbiota in beef bulls. (B) Heatmap showing the 50 most abundant ASVs (log_4_ transformed) overall in the seminal and fecal microbiota of beef bulls by sampling time and diet (H, high; M, moderate rate of gain).

To identify how the seminal microbiota differs from the microbial community residing within the cattle gut, we determined the number of unique and shared ASVs among seminal and fecal samples (Fig. 3). As expected, each anatomical site had a distinct microbiota (*R*^2^ = 0.51, *P < *0.001) (see Fig. S1 in the supplemental material), and the overall composition of the seminal microbiota differed significantly from that of the fecal microbiota. The proportion of unique ASVs in fecal and seminal microbiota accounted for 65.3% and 28.6% of the total number of ASVs (17,158), respectively (Fig. 3A). As shown in the heatmap of the 50 most abundant ASVs (Fig. 3B), there was considerable interindividual variation in both the prevalence and abundance of most of these taxa. The majority of ASVs assigned to the genera *Oceanivirga*, *Fusobacterium*, *Porphyromonas*, and *Corynebacterium* were exclusively associated in greater abundance with the seminal samples. Some taxa, however, had increased dominance in fecal samples, including *Succinivibrio* ASV27 and ASV7, *Rikenellaceae* RC9 group (ASV49 and 47), *Prevotellaceae* ASV52, and *Prevotella* ASV30.

Although the seminal and fecal microbiota were clearly distinct, a small number of taxa were present in the majority of all samples. A total of 1,053 ASVs (6.1% of total ASVs) were shared between seminal and fecal microbiota, and 7 ASVs were present in at least 90% of all fecal and seminal samples. These ASVs included ASV15 (Bifidobacterium pseudolongum), ASV16 (Romboutsia ilealis), ASV18 (*Clostridium sensu stricto* 1), ASV48 (Corynebacterium marinum), ASV65 (*Romboutsia*), ASV54 (*Paeniclostridium*), ASV120 (*Syntrophococcus*), ASV65 (*Romboutsia*), and ASV54 (*Paeniclostridium*). There were 78 ASVs that were shared by more than 60% of all samples (data not shown) and 17 ASVs that were found in at least 70% of the samples (Table 2). Two bacterial ASVs (ASV18 and ASV15) were detected in the microbiota of all fecal and semen samples. These findings suggest that a “core taxa” may exist within the bull urogenital and gastrointestinal tracts.

**TABLE 2:** Core amplicon sequence variants (ASVs) identified in at least 70% of seminal and fecal microbiota samples from yearling beef bulls^*a*^

ASV	Taxonomic assignment	ASVs identified at % of:						
		70	75	80	85	90	95	100
ASV121	Corynebacterium efficiens							
ASV181	Dietzia maris							
ASV62	Corynebacterium xerosis							
ASV48	Corynebacterium marinum							
ASV196	Bifidobacterium merycicum							
ASV198	Olsenella umbonata							
ASV143	*Ruminococcus* sp.							
ASV120	*Syntrophococcus* sp.							
ASV89	*Romboutsia* sp.							
ASV77	Blautia obeum							
ASV65	*Romboutsia* sp.							
ASV61	*Oscillospiraceae* UCG-005							
ASV54	*Paeniclostridium* sp.							
ASV37	Faecalibacterium prausnitzii							
ASV18	*Clostridium sensu stricto* 1							
ASV16	Romboutsia ilealis							
ASV15	Bifidobacterium pseudolongum							

a Shading represents the presence of the ASV at % of seminal and fecal microbiota samples.

### Total bacteria in seminal and fecal microbiota determined by quantitative PCR.

**FIG 4:**
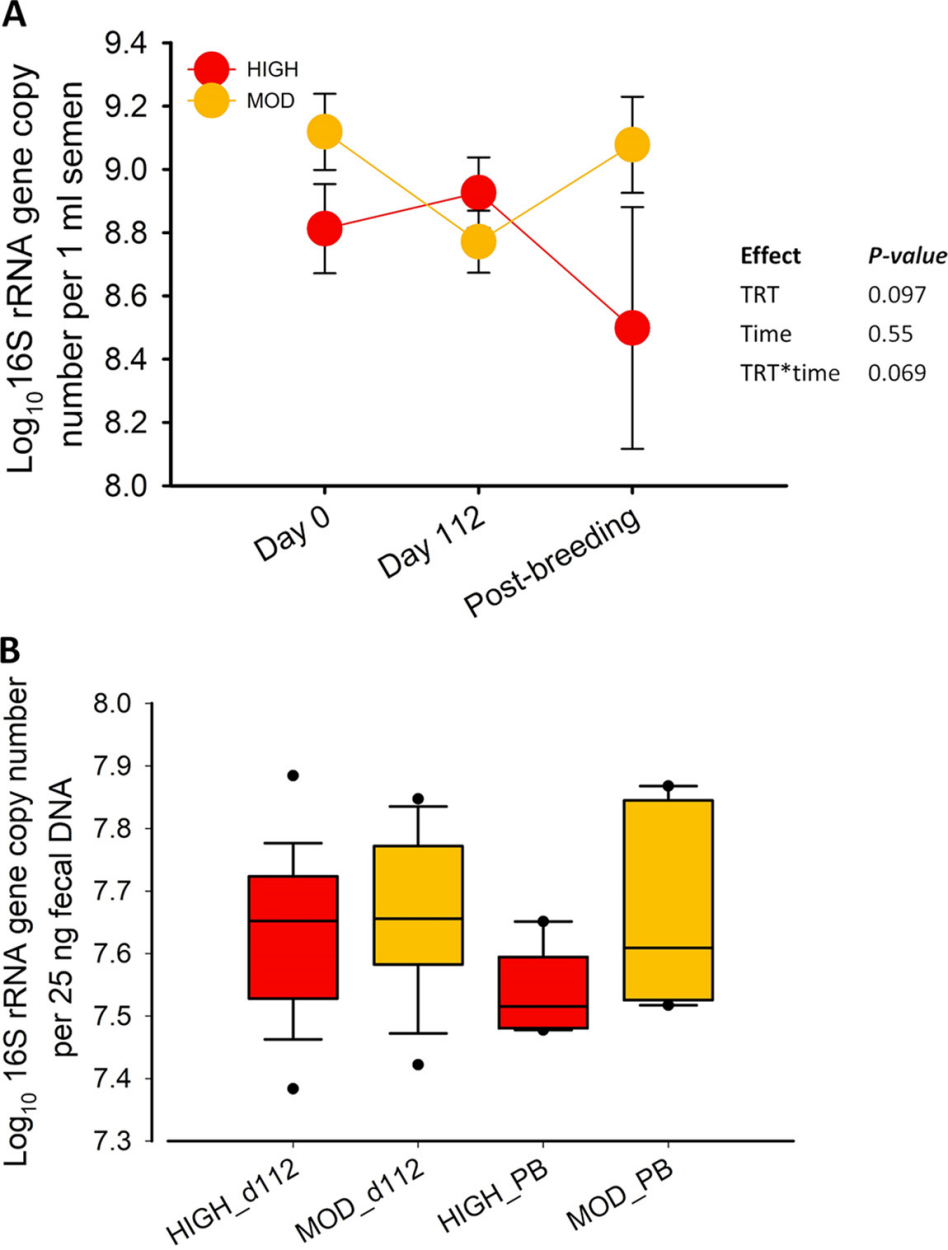
Total bacterial abundance in seminal (A) and fecal (B) samples estimated by qPCR in beef bull subjected to high (high) or moderate (moderate) rate of gains.

Total bacterial concentrations in semen samples from moderate and high bulls were 8.8 ± 0.09 and 9.0 ± 0.11 log_10_ 16S rRNA gene copies per mL semen, respectively (Fig. 4A). The total concentration of bacteria in the semen was not affected by time or dietary treatment, nor was there an interaction between time and treatment (*P > *0.05). Likewise, the total bacterial abundance in the feces of moderate and high bulls was not different at any sampling time point (*P > *0.05) (Fig. 4B).

### Bovine seminal bacterial isolates.

**(i) Aerobic culturing.** A total of 48 semen samples from day 112 (*n* = 38) and postbreeding (*n* = 10) were plated onto both *Lactobacillus* de Man, Rogosa, and Sharpe (MRS) agar and Columbia blood agar plates supplemented with 5% sheep’s blood (CB) to isolate and identify viable bacteria (Table 3). There were 57 isolates recovered from MRS plates representing 18 different genera within the *Firmicutes* (69%), *Proteobacteria* (26%), and *Actinobacteriota* (5%) phyla. An additional 163 isolates were recovered from CB plates. These isolates were classified into 29 different genera within the *Firmicutes* (56%), *Proteobacteria* (25%), *Actinobacteriota* (18%), and *Bacteroidota* (1%) phyla. Overall, 220 aerobic isolates were recovered representing 38 different genera within the *Firmicutes* (59%), *Proteobacteria* (25%), *Actinobacteriota* (15%), and *Bacteroidota* (1%) phyla. Bacteria from the *Bacillus*, Staphylococcus, *Arthrobacter*, Escherichia, and *Enterococcus* genera were most frequently isolated.

**TABLE 3:** Bacterial isolates from aerobic and anaerobic culturing of semen samples collected from yearling bulls subjected to high (high) or moderate (moderate) rates of gains^*a*^^,^^*b*^

Phylum	Genus	No. of isolates from culturing type:				Total no. of isolates
		Aerobic			Anaerobic	
		CB	MRS	Subtotal	CB	
*Firmicutes*	*Aerococcus*	ND	ND	ND	6	6
*Firmicutes*	*Bacillus*	41	13	54	29	83
*Firmicutes*	*Enterococcus*	3	11	14	21	35
*Firmicutes*	*Finegoldia*	1	ND	1	ND	1
*Firmicutes*	*Gemella*	1	ND	1	1	2
*Firmicutes*	*Hutsoniella*	ND	ND	ND	1	1
*Firmicutes*	*Kurthia*	ND	1	1	ND	1
*Firmicutes*	*Lacticaseibacillus*	1	ND	1	ND	1
*Firmicutes*	*Lactococcus*	ND	1	1	ND	1
*Firmicutes*	*Latilactobacillus*	ND	1	1	ND	1
*Firmicutes*	*Lysinibacillus*	ND	1	1	ND	1
*Firmicutes*	*Macrococcus*	ND	ND	ND	1	1
*Firmicutes*	*Niallia*	4	ND	4	3	7
*Firmicutes*	*Paenibacillus*	5	ND	5	1	6
*Firmicutes*	*Pelagirhabdus*	ND	ND	ND	1	1
*Firmicutes*	*Peptoniphilus*	ND	ND	ND	1	1
*Firmicutes*	Staphylococcus	32	6	38	8	46
*Firmicutes*	Streptococcus	2	5	7	16	23
*Firmicutes*	*Trichococcus*	ND	ND	ND	1	1
*Proteobacteria*	*Aeromonas*	1	ND	ND	ND	1
*Proteobacteria*	*Alysiella*	4	ND	4	ND	4
*Proteobacteria*	*Citrobacter*	ND	ND	ND	1	1
*Proteobacteria*	*Comamonas*	ND	1	1	ND	1
*Proteobacteria*	*Cronobacter*	1	ND	1	ND	1
*Proteobacteria*	Enterobacter	1	1	2	ND	2
*Proteobacteria*	Escherichia	10	9	19	22	41
*Proteobacteria*	*Histophilus*	ND	ND	ND	2	2
*Proteobacteria*	*Kingella*	1	ND	1	ND	1
*Proteobacteria*	*Kluyvera*	ND	ND	ND	1	1
*Proteobacteria*	*Kosakonia*	ND	ND	ND	1	1
*Proteobacteria*	*Lelliottia*	8	ND	8	2	10
*Proteobacteria*	*Mannheimia*	1	ND	1	ND	1
*Proteobacteria*	*Neisseria*	2	ND	2	ND	2
*Proteobacteria*	*Pantoea*	1	1	2	2	4
*Proteobacteria*	*Pseudescherichia*	1	ND	1	ND	1
*Proteobacteria*	Salmonella	ND	1	1	ND	1
*Proteobacteria*	*Serratia*	9	1	10	ND	10
*Proteobacteria*	*Shigella*	1	1	2	3	5
*Proteobacteria*	*Yokenella*	1	ND	1	ND	1
*Actinobacteriota*	*Actinomadura*	ND	1	1	ND	1
*Actinobacteriota*	*Arthrobacter*	26	ND	26	ND	26
*Actinobacteriota*	*Corynebacterium*	1	ND	1	ND	1
*Actinobacteriota*	*Microbacterium*	1	ND	1	ND	1
*Actinobacteriota*	*Rhodococcus*	ND	2	2	ND	2
*Actinobacteriota*	*Trueperella*	1	ND	1	1	2
*Bacteroidota*	*Algoriella*	1	ND	1	ND	1
*Bacteroidota*	*Bacteroides*	ND	ND	ND	11	11
*Bacteroidota*	*Chishuiella*	1	ND	1	ND	1
*Fusobacteriota*	*Fusobacterium*	ND	ND	ND	8	8
	Total no. of isolates	163	57	219	144	364

a Bacterial isolates were identified by nearly full-length 16S rRNA gene sequencing and BLAST. Semen samples cultured were collected on day 0, day 112, and postbreeding, and three different agar plates (CB, MRS, and blood) were used to isolate these isolates. For each isolate, the genus with the highest identity to the 16S rRNA gene sequence is listed. ND, not detected.

b Several isolates were identified as known bovine pathogens, including Fusobacterium necrophorum, Trueperella pyogenes, Mannheimia varigena, Histophilus somni, and Arthrobacter gandavensi.

**(ii) Anaerobic culturing.** A total of 144 bacterial isolates were recovered from CB agar plates incubated anaerobically (Table 3). These isolates were assigned to 24 different genera from 5 different phyla, including *Proteobacteria* (51%), *Firmicutes* (36%), *Bacteroidota* (7%) *Fusobacteriota* (5%), and *Actinobacteriota* (1%). The five most prevalent genera isolated from anaerobic culturing were *Bacillus*, Escherichia, *Enterococcus*, Streptococcus, and *Bacteroides.*

In total, 364 bacterial isolates were recovered under aerobic (*n* = 220) and anaerobic (*n* = 144) culturing conditions. Overall, 49 different genera were represented within the *Firmicutes* (60%), *Proteobacteria* (25%), *Actinobacteriota* (9%), *Bacteroidota* (4%), and *Fusobacteriota* (2%) phyla. The most prevalent genera identified with both aerobic and anaerobic culturing methods were *Bacillus*, Staphylococcus, Escherichia, *Enterococcus*, and *Arthrobacter.*

### Antimicrobial susceptibility testing of selected seminal bacterial isolates.

The MICs of 28 different antibiotics against 33 aerobic isolates (22 Gram positive and 11 Gram negative) from 16 different genera are listed in Tables 4 and 5. According to the interpretive criteria provided by CLSI M45 and M100, all Gram-positive isolates tested were susceptible to amoxicillin-clavulanate and ampicillin. Lactococcus formosensis was susceptible to all antibiotics tested except for clindamycin. Streptococcus uberis was resistant to chloramphenicol, and Bacillus mobilis, Staphylococcus haemolyticus, and Staphylococcus warneri all showed resistance to penicillin G. S. warneri was also resistant to trimethoprim-sulfamethoxazole, chloramphenicol, clindamycin, and gentamicin. Three *Enterococcus* isolates were resistant to cephalothin, cefazolin, trimethoprim-sulfamethoxazole, clindamycin, amikacin, and gentamicin.

**TABLE 4:**
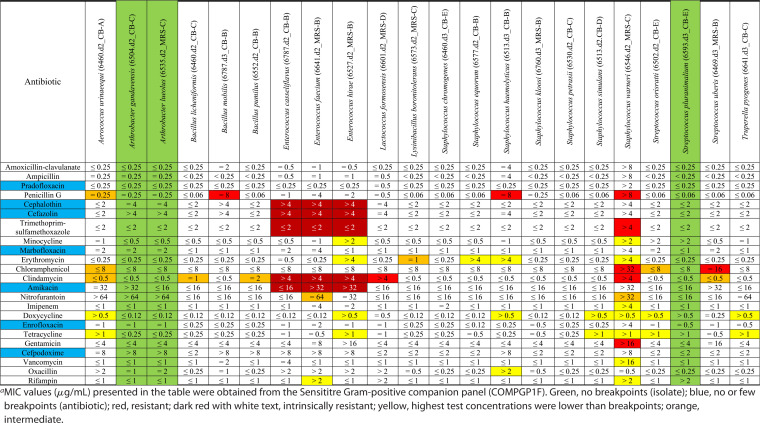
MICs of antibiotics against Gram-positive bacterial isolates (*n* = 22) isolated from the semen of beef bulls subjected to different rate of gains^*a*^

a MIC values (μg/mL) presented in the table were obtained from the Sensititre Gram-positive companion panel (COMPGP1F). Green, no breakpoints (isolate); blue, no or few breakpoints (antibiotic); red, resistant; dark red with white text, intrinsically resistant; yellow, highest test concentrations were lower than breakpoints; orange, intermediate.

**TABLE 5:**
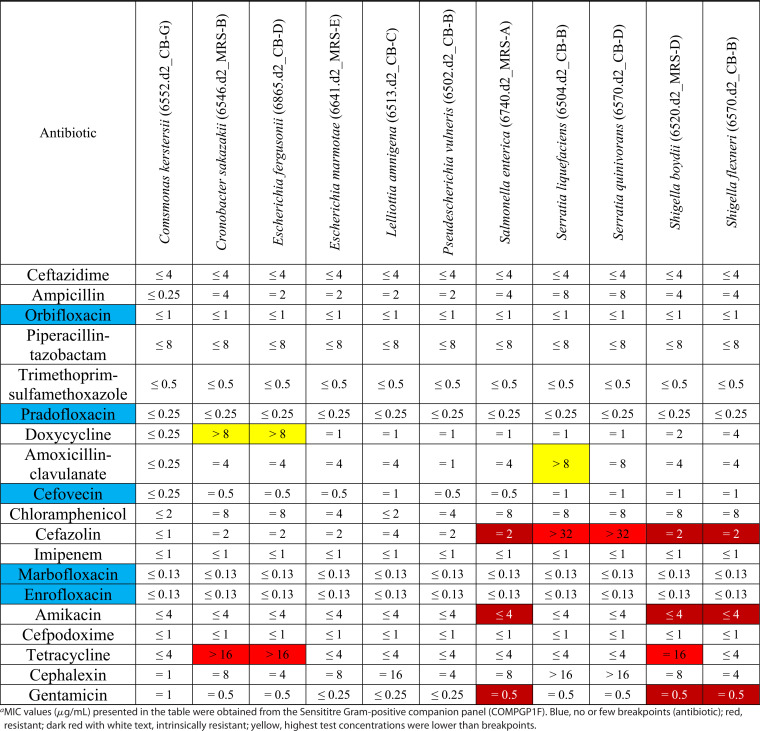
MICs of antibiotics against Gram-negative bacterial isolates (*n* = 11) isolated from the semen of beef bulls subjected to different rates of gains^*a*^

a MIC values (μg/mL) presented in the table were obtained from the Sensititre Gram-positive companion panel (COMPGP1F). Blue, no or few breakpoints (antibiotic); red, resistant; dark red with white text, intrinsically resistant; yellow, highest test concentrations were lower than breakpoints.

All Gram-negative isolates tested were susceptible to ceftazidime, ampicillin, piperacillin-tazobactam, trimethoprim-sulfamethoxazole, chloramphenicol, imipenem, cefpodoxime, and cephalexin. Serratia liquefaciens and Serratia quinivorans were resistant to cefazolin. Cronobacter sakazakii, Escherichia fergusonii, and Shigella boydii were all found to be resistant to tetracycline. There were two *Shigella* and one Salmonella isolates that were resistant to cefazolin, amikacin, and gentamicin.

There were several isolates where the resistance breakpoints were defined as greater than the concentration of the antibiotic that was tested in the Sensititre plates. Because of this, it was not possible to definitively state whether these isolates were resistant to these particular antibiotics. Three Gram-positive isolates, Arthrobacter gandavensis, Arthrobacter lueolus, and Streptococcus pluranimalium, had no available breakpoint values. Seven antibiotics that were tested on the Gram-positive panel (pradofloxacin, cephalothin, cefazolin, marbofloxacin, amikacin, enrofloxacin, and cefpodoxime) and five antibiotics from the Gram-negative panel (orbifloxacin, pradofloxacin, cefovecin, marbofloxacin, and enrofloxacin) had few to no breakpoint values available for the isolates that were tested.

## DISCUSSION

Reproductive failure remains one of the most significant problems in the beef and dairy cattle industries despite advances in genetic selection, artificial insemination, and improved feeding and management (3, 22). Emerging evidence derived from human and vertebrate animal models suggests that the microbiomes residing within the female and male urogenital tract are important for reproductive health and fertility. Therefore, manipulation of these microbiomes for improved reproductive efficiency has recently become an active area of research. While the female reproductive tract microbiome in cattle has been relatively well characterized (23–26), there is little known about the male reproductive tract microbiome. In the present longitudinal study, we characterized the seminal microbiota in yearling beef bulls fed a common diet to achieve moderate or high rates of weight gain by performing 16S rRNA gene sequencing, quantitative PCR (qPCR), (qPCR), and culturing of semen samples collected from the yearling beef bulls prior to starting a dietary treatment (day 0) and 112 days later, as well as after a 28-day breeding season. We also assessed the similarity between the seminal and fecal microbiota. Additionally, antimicrobial susceptibility testing (AST) was carried out on selected seminal isolates.

Overall, our sequencing results revealed a diverse and dynamic microbial community present in semen from yearling bulls. The seminal microbiota was dominated by members of the *Fusobacteriota*, *Bacteroidota*, *Firmicutes*, and *Actinobacteriota* phyla. Similar to our study, these four phyla have been reported to be among the dominant bacterial phyla detected in semen samples obtained from bulls that were 12 months to 6 years of age and comprised 11 different breeds (21). At the genus level, *Fusobacterium* (26%) and *Porphyromonas* (20%) were the most relatively abundant genera in the seminal microbiota of yearling bulls. Contrary to our study, Escherichia (27%) and *Bacteroides* (20%) have been reported as the most relatively abundant genera in bovine semen (21). Differences in the microbial composition of bovine seminal microbiota between our bulls and those used by Koziol and colleagues may be due to factors such as bull age, breed, feeding regimen, and housing environment. The use of different 16S rRNA primer set (V4 region primers by Koziol et al. [27] and V3-V4 primers used in our study) could attribute the difference in seminal microbiota composition between the two studies. We observed significant alterations in the microbial community structure and richness over the course of 112 days. Given that these bulls were fed the same diet, although at different rates, and were raised in the same housing environment (from day 0 to day 112), our results suggest that host age could be an important factor shaping seminal microbiota in bulls. Due to animal welfare concerns associated with collection of double-ejaculated semen samples from the same bull three times over the 112-day study period, it was not possible to evaluate the seminal microbiota from these bulls at the midpoint of the study (day 56). Further work is warranted to characterize the dynamics of the bovine seminal microbiota over time using shorter sampling intervals.

Seminal microbial community diversity and composition did not differ between the two groups at postbreeding, but there was a significant shift overall between postbreeding and day 112, suggesting dramatic changes occurred in this microbiota during the 28-day breeding season. The absence of samples from bulls not used for breeding (nonmating controls) is an acknowledged limitation of the present study, and therefore, it is challenging to establish which factors contribute to changes in the seminal microbiota during the breeding season. However, besides animal age, we argue that mating with multiple heifers on the pasture may have introduced bacterial species originating from the female reproductive tract to the bull urogenital tract and, more specifically, from the vagina. This assumption is based on the recently proposed term “complementary seminovaginal microbiota” (12); bacterial species associated with semen may have been transmitted to the vagina and/or uterus (11) and vice versa. Evidence from human studies indicated that the male and female microbiome interact with each other and that sexual intercourse is one of the most direct and significant microbial transmissions between men and women (28, 29). Accordingly, microbial transmission between the bull and heifer urogenital tracts is most likely to be the major factor attributing to the altered seminal microbiota diversity and composition that we observed after breeding. The expansive size of pastures used in the current report precluded the opportunity to evaluate individual mating activity. Also, in multiple bull-breeding pastures, females are often bred multiple times and by more than one bull (30), so identifying the number of matings based on number of calves sired is impossible. However, we conducted a retrospective genotype analysis of calves born to bulls in the current experiment and confirmed that each of the breeding bulls included in our analysis sired at least one calf (range of 1 to 46), and therefore, every single one of the bulls had successfully mated with at least one female. The effects of bull-to-female or female-to-bull microbial transfer on shaping the reproductive microbiota in cattle is also an area that warrants further research in which nonmating control bulls are compared with bulls used for breeding. This would enable to eliminate several potential confounding factors, including substantial changes in the diet (grain versus forage based) and living environments (pen versus pasture raised) before and during breeding.

Although the dietary treatment given over the course of 112 days resulted in a significantly different weight gain between the moderate and high group bulls, the seminal microbiota community structure and diversity remained unaffected by the differential weight gains achieved via a common diet. Only minor changes occurred in the seminal microbial composition in response to differential gains. Previous studies indicated that a negative plane of nutrition during spermatogenesis can have an impact on sperm motility and kinematic properties in mature beef bulls (31). Our results suggest that the microbial community associated with bovine semen is resilient to the plane of nutrition, and this might be due to the inclusion of the same dietary composition in the diet fed to both moderate and high bulls. The impact of nutrition and dietary composition on semen quality and fertility in bulls (32, 33) and humans (34) have been well documented. Thus, it is of interest to investigate whether feeding diets comprised of different grain or forage ratios can influence seminal microbiota in bulls.

The microbiota and total bacterial abundance in the feces of yearling bulls were also not influenced by differential gains achieved through different intakes of a common diet; however, the fecal microbiota underwent significant changes in terms of community structure, richness, and composition over the course of the 28-day breeding season. The major dietary shift from a corn grain-based diet to a forage-based diet on the pasture during breeding season is most likely the major factor driving the changes in the gut microbiota of the bulls observed postbreeding. Our results revealed that the seminal microbial community composition is significantly different from that of the gut microbiota, with only 6% of the ASVs shared by both microbiota (Fig. 3A) and 78% of the 50 most abundant ASVs being more exclusively present in the semen with greater abundance than feces. Likewise, the seminal and gut microbiota in humans have been reported to be very different (35). Here, we identified 17 ASVs that are part of the potential core microbiota (70% of all samples), which included genera with species with known beneficial effects on the host such as *Bifidobacterium*, *Blautia*, *Faecalibacterium*, and *Ruminococcus.* We previously reported the existence of core microbial taxa among gastrointestinal, respiratory, and female reproductive tracts in beef cattle (36). While each anatomical site of the body harbors unique and niche-specific microbiota, there is a connection between microbial communities present in the gut and other body locations and can communicate and interact with each other through secondary metabolites (37) and possibly microbial taxa shared between different sites. Thus, it is reasonable to speculate that the core taxa present in both seminal and fecal microbiota may have a potential role in mediating the communication between gut and urogenital tract microbiome in bulls. These core taxa could be transferred between the two microbial communities via internal and external routes. One of the potential internal transmission routes is blood circulation, as we know that blood-circulating microbiota exist (38). Among the external routes, mounting between the bulls could introduce urogenital tract with microbes associated with feces.

According to our aerobic and anaerobic culturing results, bovine semen is colonized by aerobic and anaerobic bacteria species largely from the *Firmicutes* (60%), *Proteobacteria* (25%), and *Actinobacteriota* (9%) phyla. When comparing the bacterial composition characterized by sequencing versus culturing methods, there is a significant discrepancy at both the phylum and genus levels. *Fusobacteriota*, which represented 36.3% of total sequencing reads, accounted for only 2% of the bacterial isolates recovered from anaerobic culturing. This may be due in part to the media used to cultivate the semen samples, as well as the strict anaerobic conditions required to grow most members of the *Fusobacteriota*. Similarly, *Bacteroidota* species were also underrepresented in culture compared to sequencing (4% versus 30.4). This might be due to that many species within the phylum *Bacteroidota* are strict anaerobes with fastidious growth requirements. Species from the *Firmicutes* and *Proteobacteria* phyla are often relatively easy to cultivate in a laboratory setting and thus could explain why there were more isolates recovered from these two phyla than the sequencing data would predict. At the genus level, *Fusobacterium* (26%) and *Porphyromonas* (20%) were the top two most relatively abundant genera identified by sequencing, whereas *Bacillus* (23%), Staphylococcus (13%), and Escherichia (11%) were the most frequently isolated genera through culturing. Despite *Fusobacterium* being the most dominant genus via sequencing, only 2% of the total bacterial isolates (*n* = 364) recovered from culturing were assigned to this genus. Freezing the semen samples prior to culturing could limit the viability and cultivability of certain bacterial species, resulting in isolation of fewer bacterial species by culturing than sequencing. The semen samples were frozen and not plated immediately upon collection due to geographical distance (approximately 900 km) between the cattle farm and the microbiology laboratory, as well as the logistical challenge and culturing consistency concerns associated with semen samples collected at three different time points (day 0, day 112, and postbreeding). To ensure the viability of the frozen semen samples, the brain heart infusion (BHI) media containing 20% glycerol was used to store the semen samples. This type of cryopreservation method has been commonly used for characterizing culturable bacteria from bovine samples (39–41). However, freeze storage can have an impact on viable bacterial cell recovery, and different bacterial species may have different tolerance to the freezing and protectant used for freezing (42). It is highly likely that the culturing results obtained from aerobic and anaerobic culturing in the present study may have underrepresent the bacteria species that are vulnerable to freeze-thaw and those that are not protected by glycerol. Overall, the discrepancy observed between the sequencing and culturing (both aerobic and anaerobic) methods highlights that most bacteria present in the bull reproductive microbial ecosystem likely require the use of several different media and growth conditions to be cultured, as well as fresh plating the semen samples if possible.

A number of seminal bacterial isolates recovered are known pathogens involved in bovine diseases. These pathogenic isolates include Fusobacterium necrophorum and Trueperella pyogenes, which are principal bacterial species associated with liver abscesses (43); Histophilus somni (bovine respiratory disease (44)), Mannheimia varigena (bovine respiratory disease and kidney infection in cattle (44, 45)), Trueperella abortisuis (46), and Bacillus cereus (47), which can cause abortion (46); Fusobacterium gastrosuis (foot rot and lameness) (48); and A. gandavensis, which has been reported to be associated with reproductive loss (49) and mastitis (50) (Table S3). The presence of these pathogenic species among the seminal microbial community begs an important question of whether bovine semen serves as a medium for transferring pathogens to female cattle through mating and, ultimately, to the offspring calves.

The emergence and spread of antimicrobial resistance (AMR) in bovine-associated pathogenic and commensal bacteria pose a serious threat to animal and public health (51, 52). Thus, monitoring and surveillance of the resistome (collection of all AMR genes associated with the microbiota in a given environment) in any microbial community are important to limit the spread of AMR in the cattle continuum (52). Therefore, we investigated whether the seminal microbiota harbors antibiotic-resistant bacteria. Our AST revealed that almost 50% of the seminal isolates tested were resistant to at least one antibiotic, and in some cases, resistance was observed for up to 6 antibiotics. These resistant isolates were comprised of both commensal and opportunistic pathogenic bacteria. Among the 33 isolates tested, 7 exhibited multidrug resistance (resistant to 3 or more antibiotics), S. warneri (penicillin G, trimethoprim-sulfamethoxazole, chloramphenicol, clindamycin, and gentamicin), 3 *Enterococcus* isolates (cephalothin, cefazolin, trimethoprim-sulfamethoxazole, clindamycin, and amikacin), 2 *Shigella* strains (cefazolin, amikacin, and gentamicin; S. boydii showed resistance against tetracycline), and Salmonella enterica (cefazolin, amikacin, and gentamicin).

Among the antibiotics that did not inhibit the growth of some seminal bacterial isolates are those that are commonly used in the cattle industry to treat bovine respiratory disease (53) and liver abscesses (tetracycline and macrolide [clindamycin]) (54), mastitis (cefazolin) (55), and metritis (penicillin) (56). Gentamicin, which is often included in extenders when preparing and storing semen for artificial insemination (AI) (57), was also found to be ineffective against several seminal isolates tested. Overall, our results suggest that antibiotic-resistant bacteria are present in the urogenital tract of bulls and that semen may transfer AMR from bulls to the female reproductive tract and may even spread AMR among female cattle during breeding, as a single bull will often mate with many females. Considering that these yearling bulls were not given antibiotics except for monensin, which was included in the diet, and had not been exposed to any females, future work should evaluate the origin of the seminal resistome and its contribution to the spread of AMR in female cattle using a metagenomic-based approach.

There are limitations associated with our study. One of the limitations was the absence of semen sampling in the middle (day 56) of the 112-day feeding period, which was due to the animal welfare concerns associated with collection of double-ejaculated semen samples. Another limitation was the lack of data from cohort bulls not used for breeding (nonmating controls). This was due to the logistical and financial challenges. The third limitation was associated with freezing the semen samples prior to the culturing, which might have limited the recovery of certain bacterial species. Despite these limitations, there are several strengths in our study compared to the other studies on the bull seminal microbiota published so far (58). The semen samples used in our study were from the same bulls collected at three different time points, which represent different bull growth and developmental stages, and from three full spermatogenic cycles, as well as pre- and postbreeding stages, providing novel and important information on how the seminal microbial community changes with age and is influenced by breeding activity. The genetic background, age, and body weight of all bulls were significantly homogenous, which makes our study unique and superior to the other studies in which the semen was collected at a single time point and from bulls with different ages, different genetic backgrounds, and different farms and were sampled at different seasons. Despite our data being limited to the taxonomic characterization of bovine seminal microbiota, our study provides strong evidence of the presence of bovine seminal microbiota, its taxonomic composition (culturable and nonculturable), factors shaping this microbial community, and its dissimilarity with bovine fecal microbiota. These insights will serve as an important basis for the future understanding of the functional features and the role of the seminal microbiome in defining reproductive health and fertility in cattle.

### Conclusions.

Yearling bull semen contains a rich and complex microbiota, which changes as bulls age and during the breeding season while remaining unaffected by differential weight gains achieved via a common diet. The taxonomic composition of the seminal microbiota was distinct from the fecal microbiota, with only 17 ASVs identified as core taxa shared between the two communities. The fecal microbiota of bulls was not influenced by differential weight gain but was significantly altered following the 28-day breeding season. Our culturing results revealed that bovine semen harbors not only commensal bacteria but also potential bacterial pathogens associated with bovine respiratory disease, liver abscesses, reproductive infections, mastitis, and lameness in cattle. We also identified antibiotic-resistant bacteria present in bovine semen. Overall, our results obtained from 16S rRNA gene sequencing, anaerobic and aerobic culturing, as well as antibiotic susceptibility testing, provide novel insights into the seminal microbiota of yearling beef bulls.

## MATERIALS AND METHODS

All experimental procedures involving cattle were approved by the USDA, Agriculture Research Service, U.S. Meat Animal Research Center Institutional Animal Care and Use Committee (USMARC IACUC; experiment number 147.2).

### Animal husbandry and experimental design.

**FIG 5:**
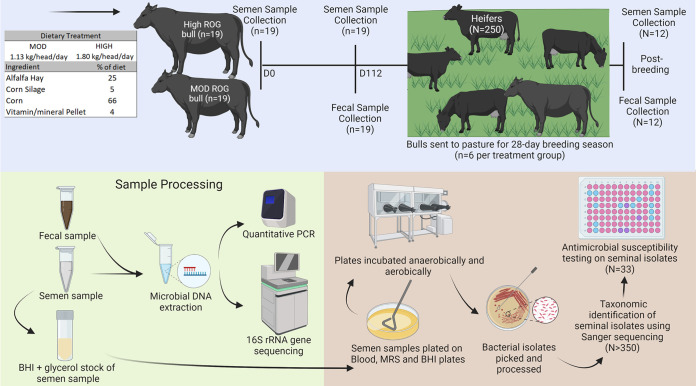
Schematic overview of the study design, sampling regimen, and sample processing.

Forty Marc II (composite, one-quarter Angus, one-quarter Hereford, one-quarter Gelbvieh. one-quarter Simmental) yearling bulls (initial body weight [BW], 503 ± 7.2 kg) were selected from the year 2020 calving season. These bulls were 9 to 10 months of age, averaged a body condition score of 5 to 5.5, and had not been previously exposed to any females. Bulls were stratified to treatment based on pubertal status and initial weight and housed in three (15, 15, and 10) partially covered pens. All bulls were individually fed with Calan gates (American Calan, Northwood, NH, USA), which were on a cement-covered pad. The bulls were trained to use the Calan gates for at least 4 weeks. Bulls had *ad libitum* access to a total mixed ration containing 25% alfalfa hay, 5% corn silage, 66% corn, and 4% vitamin/mineral pellet (dry matter basis). After the training period was over, two bulls that failed to train to the Calan gates were removed from the study. The remaining bulls were randomly assigned to one of the two intake regimens of the same ration to achieve moderate (1.13 kg/head/day; moderate) or high (1.80 kg/head/day; high) rates of weight gain (as shown in the schematic overview of the study design and sampling regimen in Fig. 5). The bulls were fed for 112 days (February to May 2021) and weighed frequently prior to feed delivery to ensure that the bulls were meeting their targeted rates of gain, and diet delivery was adjusted biweekly to achieve targeted gains. Feed was provided to the bulls once daily at 0800.

On day 112, six bulls from each treatment group who passed their breeding soundness examination (BSE) were randomly selected for use in breeding. Breeding soundness examinations include a physical evaluation, measurement of scrotal circumference, and an evaluation of semen motility and morphology (27).

These selected bulls were put in a pasture with 250 commercial red heifers for a 28-day breeding season (10 June 2021 to 7 July 2021). After the breeding season was over, these 12 bulls were subjected to BSE to examine postbreeding semen quality. Of note, the 28-day breeding period used is the standard breeding season for the replacement heifers at the U.S. Meat Animal Research Center (USMARC) cattle farm, and a shorter breeding season (28 days) for replacement heifers than a more traditional 45- to-60-day breeding season will pay dividends in long future (59, 60). The number of bulls (*n* = 12) used for breeding was based on the yearling bull-to-heifer mating ratio (1 bull per 21 heifers). This ratio was slightly less than the typical yearling bull/heifer ratio (1:25) in a commercial operation (61), as the possibility of removing bulls during breeding due to illness or other reasons was considered.

### Seminal and fecal sampling.

Semen was collected on days 0 and 112 (*n* = 19 per treatment group) and postbreeding (*n* = 6 per treatment group) using electroejaculation (Pulsator IV; Lane Manufacturing Inc.; Denver, CO) by a veterinarian according to the methods described previously (62) (Fig. 5). Two sampling time points (days 0 and 112) were chosen for semen collection, as a third sampling time at study midpoint (day 56) was not permitted due to animal welfare concerns (double ejaculation was needed at each sampling time point). Also, semen sample collection on day 112 allows almost two full spermatogenic cycles in bulls and for adequate weight separation between bulls at the moderate rate of gain and those at the high rate of gain. Bulls were an average of 303 days (minimum, 281 days; maximum, 329 days) old at day 0 sampling. Disposable plastic bags manufactured for semen collection were used. A 200-μL aliquot of semen was placed into 1-mL brain heart infusion (BHI) broth with 20% glycerol and immediately placed on dry ice. Another 200-μL aliquot of semen was stored in a 1.5-mL centrifuge tube for genomic DNA extraction.

Fecal samples were collected prior to semen collection on day 112 and postbreeding with a long-sleeve glove without lubrication via the rectal grab technique. Approximately 100 to 150 g of the fecal grab sample was transferred from the glove into a sterile Whirl-Pak sampling bag and immediately frozen on dry ice. Semen and fecal samples were transported from the farm to the lab on dry ice and then stored at −80°C until DNA extraction and culturing (semen samples). All samples were collected in the same indoor cattle handling facility by the same personnel from February 2021 to July 2021.

### Metagenomic DNA extraction.

**(i) Seminal DNA extraction.** The DNeasy blood and tissue kit (Qiagen Inc., Hilden, Germany) was used to extract DNA from semen samples. Prior to extraction, 50 μL of whole semen was dispensed into a 2-mL screw cap tube and centrifuged at 20,000 × *g* for 10 min. The supernatant was discarded, and the pellet was thoroughly resuspended with 140 μL of enzymatic lysis buffer (20 mM Tris-HCl, pH 8, 2 mM sodium EDTA, 1.2% Triton X-100, lysozyme [20 mg/mL], and mutanolysin [300 U/mL]) and then incubated for 1 h at 37°C with agitation at 800 rpm (36). The DNeasy blood and tissue kit was then used as described in the manufacturer’s protocol with the addition of Tris(2-carboxyethyl)phosphine hydrochloride (TCEP) solution (Thermo Scientific, Waltham, MA, USA) to the sample for a final concentration of 50 mM prior to bead beating. The DNA was stored at −20°C until it was used for 16S rRNA gene sequencing and estimation of total bacterial abundance using qPCR. Of note, enzymatic lysis and bead beating were added to the DNA extraction procedure to effectively lyse cells and maximize the DNA extraction from both Gram-positive and Gram-negative species for bacterial community analysis (63).

**(ii) Fecal DNA extraction.** The DNeasy Powerlyzer PowerSoil kit (Qiagen Inc.) was used to extract DNA from 200 to 250 mg of fecal material from each bull according to the manufacturer’s instructions. The concentration of extracted DNA was measured using a NanoDrop ND-1000 spectrophotometer and PicoGreen. DNA was stored at −20°C until it was used for 16S rRNA gene sequencing and estimation of total bacterial abundance using qPCR.

**(iii) 16S rRNA gene sequencing and analysis.** The V3-V4 hypervariable regions of the 16S rRNA gene were amplified using the 341-F (5′-CCTAYGGGRBGCASCAG-3′) and 806-R (5′-GGACTACNNGGGTATCTAAT-3′) primers as described previously (36). All PCR steps were carried out using the Phusion High-Fidelity PCR master mix (New England Biolabs, Ipswich, MA, USA). The PCR products were electrophoresed on a 2% agarose gel and stained with SYBR Safe DNA gel stain. The DNA fragment was excised from the gel and purified using the QIAquick gel extraction kit (Qiagen Inc.). Sequencing libraries were generated with the NEBNext Ultra DNA library prep kit (New England Biolabs) for Illumina, following the recommendations of the manufacturer. Library quality was assessed with a Qubit 2.0 fluorometer (Thermo Scientific) and Agilent Bioanalyzer 2100 system. Libraries were then sequenced on a NovaSeq 6000 instrument with an SP flow cell (2 × 250 bp) (Illumina Inc., San Diego, CA, USA).

The 16S rRNA gene sequences were processed using DADA2 v.1.18 (64) in R v.4.0.3. Briefly, the forward reads were truncated at 225 bp and the reverse reads at 220 bp. The reads were merged, chimeric sequences removed, and taxonomy assigned to each merged sequence (amplicon sequence variant [ASV]), using the naive Bayesian RDP classifier (65) and the SILVA SSU database release 138.1 (66). The ASVs that were predominantly in the negative extraction control samples and likely to be contaminants were removed prior to analyses, as were those ASVs classified as chloroplasts, mitochondria, or eukaryota. The number of ASVs per sample (richness), the Shannon and inverse Simpson’s diversity indices, and Bray-Curtis dissimilarities were calculated in R using phyloseq 1.34.0 (67) and vegan 2.5 to 7 (68). To account for uneven sequence depths, samples were randomly subsampled to 39,000 and 52,000 for the seminal and fecal samples, respectively, prior to the calculation of Bray-Curtis dissimilarities and diversity measures.

### Bacterial concentration in seminal and fecal samples.

Total bacterial load was estimated from seminal and fecal samples using real-time qPCR. The primers 515F (5′-GTGYCAGCMGCCGCGGTAA-3′) and 806R (5′-GGACTACNVGGGTWTCTAAT-3′) were used to amplify the V4 region of the 16S rRNA gene as described previously (69, 70).

### Isolation of bacteria from semen samples using anaerobic and aerobic culturing.

In addition to sequencing, an extensive culturing (both aerobic and anaerobic) was applied to characterize cultivable fraction of the seminal microbiota and to complement the 16S rRNA gene sequencing results by providing higher taxonomic resolution.

### (i) Aerobic culturing.

Fifty-microliter aliquots of semen preserved in BHI plus 20% glycerol from day 112 (*n* = 38) and postbreeding (*n* = 10) samples were spread onto both *Lactobacillus* De Man, Rogosa, and Sharpe (MRS) agar and Columbia blood agar plates supplemented with 5% sheep’s blood (CB) and incubated at 37°C in 5% CO_2_ for up to 48 h. Up to eight colonies with distinctive morphologies on each plate were then subcultured onto their respective agar and incubated under the same conditions described above. After visually assessing the purity of each isolate, a disposable loop was used to transfer each isolate into 100 μL Tris-EDTA (TE) stock and 1 mL of 20% (vol/vol) glycerol containing MRS or BHI broth, depending on what agar the isolate was grown on. The TE stocks were stored at −20°C for genomic DNA extraction, while the MRS (MRSg) and BHI glycerol (BHIg) stocks were stored at −80°C.

### (ii) Anaerobic culturing.

Anaerobic culturing was performed in an anaerobic chamber (type B, vinyl; Coy Laboratory Products Inc., Grass Lake, MI). Fifty-microliter aliquots of seminal BHI glycerol stock from day 112 (*n* = 38) and postbreeding (*n* = 10) samples were plated onto CB agar and incubated at 37°C for 48 h. Colonies with a unique morphology were selected and subcultured onto a CB plate and incubated at 37°C for 24 h. A loopful of each isolate was transferred to 100 μL TE and 1 mL BHI glycerol stock and then stored at −20°C or −80°C, respectively, until used.

### Identification of seminal isolates.

Genomic DNA was extracted from all MRS (*n* = 57) and CB agar (aerobic, *n* = 163; anaerobic, *n* = 144) isolates using a Quick-DNA fungal/bacterial miniprep kit (Zymo Research, Irvine, CA, USA) according to the manufacturer’s instructions with the following modifications: (i) 70-μL of TE stock was used as the input material, (ii) samples were processed at 4.5 m/s for 30 s in an MP FastPrep-24 bead beater (MP Biomedicals, Irvine, CA, USA), and (iii) DNA was eluted with 40 μL of elution buffer. The extracted DNA was stored at −20°C until it was needed for PCR.

Nearly the full length of the 16S rRNA gene was amplified via PCR using the universal primers 27F (5′-AGAGTTTGATCMTGGCTCAG-3′) and 1492R (5′-TACGGYTACCTTGTTACGACTT-3′). Each PCR consisted of 20-μL iQ supermix (Bio-Rad Laboratories, Inc., Hercules, CA, USA) and 1 μL of each primer (10 μM), and 2 μL of isolate DNA for a total volume of 40 μL per reaction. The PCR conditions were as follows: an initial denaturation of 95°C for 5 min; 35 cycles of 95°C for 45 s, 50°C for 30 s, and 72°C for 2 min; and a final extension at 72°C for 5 min. The PCRs were performed using an Eppendorf Mastercycler (Eppendorf, Hamburg, Germany). A 1% (wt/vol) agarose gel was used to visualize the PCR products, and amplicons were sent to MCLab (San Francisco, CA, USA) for Sanger sequencing. The 16S rRNA gene sequences were identified using the Basic Local Alignment Search Tool (BLAST) and the nonredundant NCBI nucleotide database.

### Evaluation of antimicrobial susceptibilities of selected seminal isolates.

A total of 33 aerobic isolates (22 Gram positive and 11 Gram negative) were randomly selected and subjected to antimicrobial susceptibility testing (AST). Fresh agar plates inoculated with MRSg or BHIg stock cultures were submitted to the North Dakota State University (NDSU) Veterinary Diagnostic Laboratory for AST. The MICs of 28 antibiotics were determined by the microdilution method (Sensititre; Thermo Fisher Scientific, Nepean, ON, Canada) using commercially available panels (companion animal Gram positive [COMPGP1F], companion animal Gram negative [COMPGN1F]; Trek Diagnostic Systems, Cleveland, OH, USA). The AST was performed according to the procedures recommended for the COMPGP1F and COMPGN1F panels. The antimicrobials tested are listed in Tables S1 and S2. Bacterial isolates were inoculated onto plates using a Sensititre AIM delivery system, and after 24 h incubation, plates were evaluated with a Biomic V3 (Giles Scientific USA; Santa Barbara, CA, USA). Quality control was performed on the microdilution plates as directed by the Clinical and Laboratory Standards Institute (CLSI) VET01S standards (71). Breakpoints for *Bacillus*, *Lysinibacillus*, and *Trueperella* isolates were determined using the criteria provided by CLSI document M45 (72). All other breakpoints were determined using CLSI M100 (Tables S1 and S2) (73).

### Antibiotic ingestion and treatment history.

The bull diet contained 200 mg monensin per bull primarily for use as a coccidiostat. No bulls were treated with antibiotics throughout the study.

### Statistical analysis.

The effect of diet and sampling time on the seminal and fecal microbial community structure was assessed using Bray-Curtis dissimilarities and PERMANOVA (adonis2 function). Pairwise comparisons of the Bray-Curtis dissimilarities among different sampling time points and treatment groups were done using the R package pairwise Adonis v. 0.01 with the Benjamini-Hochberg procedure used to correct for multiple comparisons. The number of ASVs (richness), alpha diversity indices, and relative abundance of the most relatively abundant phyla and genera between treatment groups within a sampling time point and between different sampling time points were compared using the generalized linear mixed-model estimation procedure (PROC GLIMMIX) in SAS (ver. 9.4, SAS Institute Inc., Cary, NC, USA). The model included the main effects rate of gain (high or moderate), sampling time, and the respective interactions. Means among different sampling times or treatment groups within each sample type were compared using the lsmeans statement, and significance was declared at a *P* value of <0.05.

### Data availability.

Sequencing data generated for this study is available at the NCBI Sequence Read Archive under BioProject accession no. PRJNA857693.
