# Supplementary material for: A Longitudinal Characterization of the Seminal Microbiota and Antibiotic Resistance in Yearling Beef Bulls Subjected to Different Rates of Gain
Source: Microbiol Spectr. 2023 Mar 14;11(2):e05180-22. doi: 10.1128/spectrum.05180-22 (PMC10100376; doi:10.1128/spectrum.05180-22)
Supplement: Supplemental file 1 — Supplemental material. Download spectrum.05180-22-s0001.pdf, PDF file, 0.3 MB [file spectrum.05180-22-s0001.pdf]

Supplemental material FOR publication.

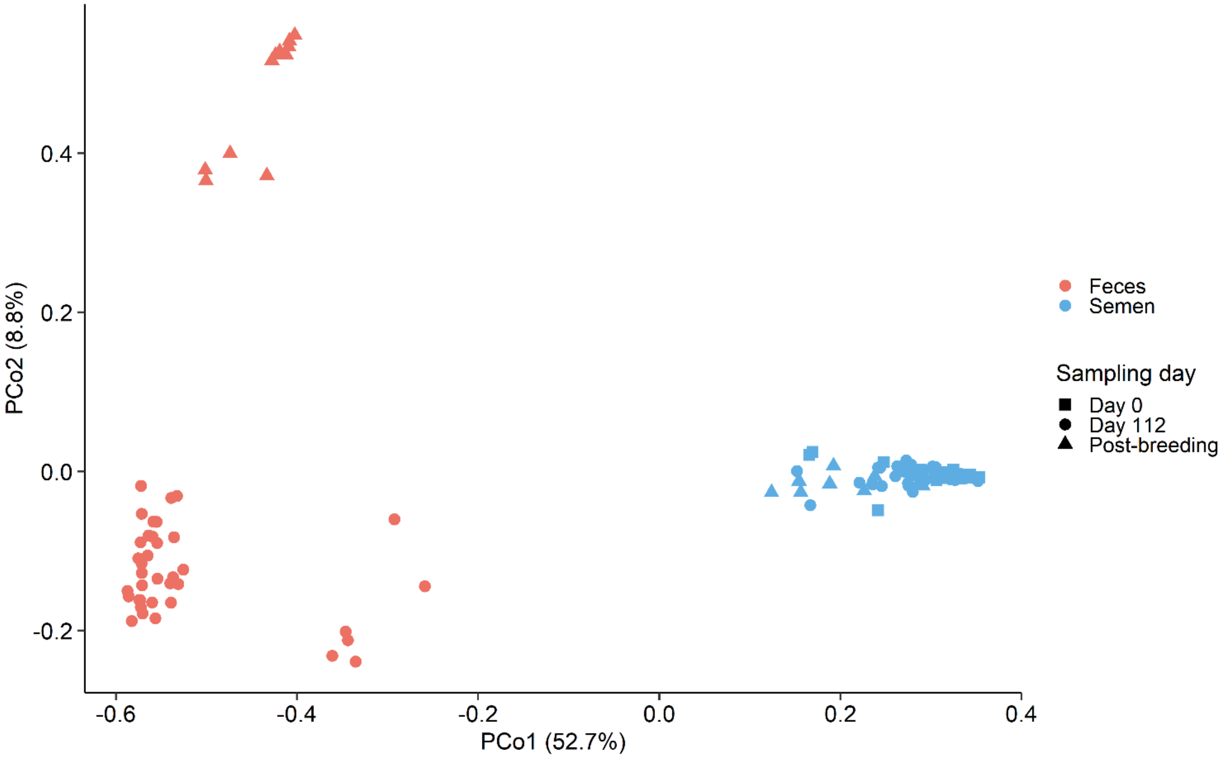

Supplementary Figure 1. Principal coordinates analysis (PCoA) plot of the Bray–Curtis dissimilarities of the seminal and fecal microbiota of yearling beef bulls (PERMANOVA, R^2 = 0.51; $P < 0.001$).

Supplementary Table 1a. Antimicrobial resistance breakpoints that have been reported previously (Gram-positive; (37, 38)

Antibiotic	*Aerococcus urinaeequi(*	*Arthrobacter gandavensis*	*Arthrobacter lueolus*	*Bacillus licheniformis*	*Bacillus mobilis*	*Bacillus pumilus*	*Enterococcus casseliflavus*	*Enterococcus faecium*	*Enterococcus hirae*	*Lactococcus formosensis*	*Lysinibacillus boronitolerans*
Amoxicillin-clavlanate	-	-	-	-	-	-	≤ 8/-/≥ 16	≤ 8/-/≥ 16	≤ 8/-/≥ 16	-	-
Ampicillin	≤ 0.25/0.5-4/≥ 8	-	-	≤ 0.25/-/0.5	≤ 0.25/-/0.5	≤ 0.25/-/0.5	≤ 8/-/≥ 16	≤ 8/-/≥ 16	≤ 8/-/≥ 16	≤ 1/2/≥ 4	≤ 0.25/-/0.5
Pradofloxacin	-	-	-	-	-	-	-	-	-	-	-
Penicillin G	≤ 0.12/0.25-2/≥ 4	-	-	≤ 0.12/-/≥ 0.25	≤ 0.12/-/≥ 0.25	≤ 0.12/-/≥ 0.25	≤ 8/-/≥ 16	≤ 8/-/≥ 16	≤ 8/-/≥ 16	≤ 1/2/≥ 4	≤ 0.12/-/≥ 0.25
Cephalothin	-	-	-	-	-	-	-	-	-	-	-
Cefazolin	-	-	-	-	-	-	-	-	-	-	-
Trimethoprim-sulfamethoxazole	-	-	-	≤ 2/-/≥ 4	≤ 2/-/≥ 4	≤ 2/-/≥ 4	-	-	-	≤ 2/-/≥ 4	≤ 2/-/≥ 4
Minocycline	≤ 2/4/≥ 8	-	-	-	-	-	≤ 4/8/≥ 16	≤ 4/8/≥ 16	≤ 4/8/≥ 16	-	-
Marbofloxacin	-	-	-	-	-	-	-	-	-	-	-
Erythromycin	≤ 0.25/0.5/≥ 1	-	-	≤ 0.5/1-4/≥ 8	≤ 0.5/1-4/≥ 8	≤ 0.5/1-4/≥ 8	≤ 0.5/1-4/≥ 8	≤ 0.5/1-4/≥ 8	≤ 0.5/1-4/≥ 8	≤ 0.5/1-4/≥ 8	≤ 0.5/1-4/≥ 8
Chloramphenicol	≤ 4/8/≥ 16	-	-	≤ 8/16/≥ 32	≤ 8/16/≥ 32	≤ 8/16/≥ 32	≤ 8/16/≥ 32	≤ 8/16/≥ 32	≤ 8/16/≥ 32	-	≤ 8/16/≥ 32
Clindamycin	≤ 0.25/0.5/≥ 1	-	-	≤ 0.5/1-2/≥ 4	≤ 0.5/1-2/≥ 4	≤ 0.5/1-2/≥ 4	-	-	-	≤ 0.5/1-2/≥ 4	≤ 0.5/1-2/≥ 4
Amikacin	-	-	-	≤ 16/32/≥ 64	≤ 16/32/≥ 64	≤ 16/32/≥ 64	-	-	-	-	≤ 16/32/≥ 64
Nitrofurantoin	-	-	-	-	-	?	≤ 32/64/≥ 128	≤ 32/64/≥ 128	≤ 32/64/≥ 128	-	-
Imipenem	-	-	-	≤ 4/8/≥ 16	≤ 4/8/≥ 16	≤ 4/8/≥ 16	-	-	-	-	-
Doxycycline	≤ 2/4/≥ 8	-	-	-	-	-	≤ 4/8/≥ 16	≤ 4/8/≥ 16	≤ 4/8/≥ 16	-	-
Enrofloxacin	-	-	-	-	-	-	-	-	-	-	-
Tetracycline	≤ 2/4/≥ 8	-	-	≤ 4/8/≥ 16	≤ 4/8/≥ 16	≤ 4/8/≥ 16	≤ 4/8/≥ 16	≤ 4/8/≥ 16	≤ 4/8/≥ 16	≤ 2/4/≥ 8	≤ 4/8/≥ 16
Gentamicin	-	-	-	≤ 4/8/≥ 16	≤ 4/8/≥ 16	≤ 4/8/≥ 16	-	-	-	-	≤ 4/8/≥ 16
Cefpodoxime	-	-	-	-	-	-	-	-	-	-	-
Vancomycin	≤ 1/-/-	-	-	≤ 4/-/-	≤ 4/-/-	≤ 4/-/-	≤ 4/8-16/≥ 32	≤ 4/8-16/≥ 32	≤ 4/8-16/≥ 32	≤ 2/-/-	≤ 4/-/-
Oxacillin	-	-	-	-	-	-	-	-	-	-	-
Rifampin	-	-	-	≤ 1/2/≥ 4	≤ 1/2/≥ 4	≤ 1/2/≥ 4	≤ 1/2/≥ 4	≤ 1/2/≥ 4	≤ 1/2/≥ 4	-	≤ 1/2/≥ 4

Supplementary Table 1 cont'd. Antimicrobial resistance breakpoints that have been reported (Gram-positive; (37, 38)

Antibiotic	*Staphylococcus chromogenes*	*Staphylococcus equorum*	*Staphylococcus haemolyticus*	*Staphylococcus kloosi*	*Staphylococcus petrasii*	*Staphylococcus simulans*	*Staphylococcus warneri*	*Streptococcus orisratti*	*Streptococcus pluranimalium*	*Streptococcus uberis*	*Truperella pyogenes*
Amoxicillin-clavlanate	-	-	-	-	-	-	-	≤ 0.25/-/-	-	-	-
Ampicillin	-	-	-	-	-	-	-	≤ 0.25/-/-	-	≤ 0.25/0.5-4/≥ 8	-
Pradofloxacin	-	-	-	-	-	-	-	-	-	-	-
Penicillin G	≤ 0.12/-/≥ 0.25	≤ 0.12/-/≥ 0.25	≤ 0.12/-/≥ 0.25	≤ 0.12/-/≥ 0.25	≤ 0.12/-/≥ 0.25	≤ 0.12/-/≥ 0.25	≤ 0.12/-/≥ 0.25	≤ 0.12/-/-	-	≤ 0.12/0.25-2/≥ 4	≤ 0.12/0.25-2/≥ 4
Cephalothin	-	-	-	-	-	-	?	≤ 0.12/-/-	-	-	-
Cefazolin	-	-	-	-	-	-	?	≤ 0.12/-/-	-	-	-
Trimethoprim-sulfamethoxazole	≤ 2/-/≥ 4	≤ 2/-/≥ 4	≤ 2/-/≥ 4	≤ 2/-/≥ 4	≤ 2/-/≥ 4	≤ 2/-/≥ 4	≤ 2/-/≥ 4	-	-	-	≤ 2/-/≥ 4
Minocycline	≤ 4/8/≥ 16	≤ 4/8/≥ 16	≤ 4/8/≥ 16	≤ 4/8/≥ 16	≤ 4/8/≥ 16	≤ 4/8/≥ 16	≤ 4/8/≥ 16	-	-	≤ 2/4/≥ 8	-
Marbofloxacin	-	-	-	-	-	-	-	-	-	-	-
Erythromycin	≤ 0.5/1-4/≥ 8	≤ 0.5/1-4/≥ 8	≤ 0.5/1-4/≥ 8	≤ 0.5/1-4/≥ 8	≤ 0.5/1-4/≥ 8	≤ 0.5/1-4/≥ 8	≤ 0.5/1-4/≥ 8	≤ 0.25/0.5/≥ 1	-	≤ 0.25/0.5/≥ 1	≤ 0.5/1/≥ 2
Chloramphenicol	≤ 8/16/≥ 32	≤ 8/16/≥ 32	≤ 8/16/≥ 32	≤ 8/16/≥ 32	≤ 8/16/≥ 32	≤ 8/16/≥ 32	≤ 8/16/≥ 32	≤ 4/8/≥ 16	-	≤ 4/8/≥ 16	-
Clindamycin	≤ 0.5/1-2/≥ 4	≤ 0.5/1-2/≥ 4	≤ 0.5/1-2/≥ 4	≤ 0.5/1-2/≥ 4	≤ 0.5/1-2/≥ 4	≤ 0.5/1-2/≥ 4	≤ 0.5/1-2/≥ 4	-	-	≤ 0.25/0.5/≥ 1	≤ 0.5/1-2/≥ 4
Amikacin	-	-	-	-	-	-	-	-	-	-	-
Nitrofurantoin	≤ 32/64/≥ 128	≤ 32/64/≥ 128	≤ 32/64/≥ 128	≤ 32/64/≥ 128	≤ 32/64/≥ 128	≤ 32/64/≥ 128	≤ 32/64/≥ 128	-	-	-	-
Imipenem	-	-	-	-	-	-	-	≤ 0.12/-/-	-	-	-
Doxycycline	≤ 4/8/≥ 16	≤ 4/8/≥ 16	≤ 4/8/≥ 16	≤ 4/8/≥ 16	≤ 4/8/≥ 16	≤ 4/8/≥ 16	≤ 4/8/≥ 16	-	-	≤ 2/4/≥ 8	≤ 4/8/≥ 16
Enrofloxacin	?	?	?	?	?	?	?	?	-	-	-
Tetracycline	≤ 4/8/≥ 16	≤ 4/8/≥ 16	≤ 4/8/≥ 16	≤ 4/8/≥ 16	≤ 4/8/≥ 16	≤ 4/8/≥ 16	≤ 4/8/≥ 16	≤ 2/4/≥ 8	-	≤ 2/4/≥ 8	≤ 4/8/≥ 16
Gentamicin	≤ 4/8/≥ 16	≤ 4/8/≥ 16	≤ 4/8/≥ 16	≤ 4/8/≥ 16	≤ 4/8/≥ 16	≤ 4/8/≥ 16	≤ 4/8/≥ 16	?	-	-	≤ 4/8/≥ 16
Cefpodoxime	-	-	-	-	-	-	-	-	-	-	-
Vancomycin	≤ 4/8-16/≥ 32	≤ 4/8-16/≥ 32	≤ 4/8-16/≥ 32	≤ 4/8-16/≥ 32	≤ 4/8-16/≥ 32	≤ 4/8-16/≥ 32	≤ 4/8-16/≥ 32	≤ 1/-/-	-	≤ 1/-/-	≤ 2/-/-
Oxacillin	≤ 2/-/≥ 4	≤ 2/-/≥ 4	≤ 2/-/≥ 4	≤ 2/-/≥ 4	≤ 2/-/≥ 4	≤ 2/-/≥ 4	≤ 2/-/≥ 4	-	-	-	-
Rifampin	≤ 1/2/≥ 4	≤ 1/2/≥ 4	≤ 1/2/≥ 4	≤ 1/2/≥ 4	≤ 1/2/≥ 4	≤ 1/2/≥ 4	≤ 1/2/≥ 4	-	-	-	≤ 1/2/≥ 4

Supplementary Table 2: Antimicrobial resistance breakpoints that have been reported previously (Gram-negative;(37, 38)

Antibiotic	*Comsmonas kerstersii*	*Cronobacter sakazakii*	*Escherichia fergusonii*	*Escherichia marmotae*	*Lelliottia amnigena*	*Pseudescherichia vulneris*	*Salmonella enterica*	*Serratia liquefaciens*	*Serratia quinivorans*	*Shigella boydii*	*Shigella flexneri*
Ceftazidime	≤ 8/16/≥ 32	≤ 4/8/≥ 16	≤ 4/8/≥ 16	≤ 4/8/≥ 16	≤ 4/8/≥ 16	≤ 4/8/≥ 16	≤ 4/8/≥ 16	≤ 4/8/≥ 16	≤ 4/8/≥ 16	≤ 4/8/≥ 16	≤ 4/8/≥ 16
Ampicillin	-	≤ 8/16/≥ 32	≤ 8/16/≥ 32	≤ 8/16/≥ 32	≤ 8/16/≥ 32	≤ 8/16/≥ 32	≤ 8/16/≥ 32	≤ 8/16/≥ 32	≤ 8/16/≥ 32	≤ 8/16/≥ 32	≤ 8/16/≥ 32
Orbifloxacin	-	-	-	-	-	-	-	-	-	-	-
Piperacillin-tazobactam	≤ 16/32-64/≥ 128	≤ 8/-/≥ 32	≤ 8/-/≥ 32	≤ 8/-/≥ 32	≤ 8/-/≥ 32	≤ 8/-/≥ 32	≤ 8/-/≥ 32	≤ 8/-/≥ 32	≤ 8/-/≥ 32	≤ 8/-/≥ 32	≤ 8/-/≥ 32
Trimethoprim-sulfamethoxazole	≤ 2/-/≥ 4	≤ 2/-/≥ 4	≤ 2/-/≥ 4	≤ 2/-/≥ 4	≤ 2/-/≥ 4	≤ 2/-/≥ 4	≤ 2/-/≥ 4	≤ 2/-/≥ 4	≤ 2/-/≥ 4	≤ 2/-/≥ 4	≤ 2/-/≥ 4
Pradofloxacin	-	-	-	-	-	-	-	-	-	-	-
Doxycycline	≤ 4/8/≥ 16	≤ 4/8/≥ 16	≤ 4/8/≥ 16	≤ 4/8/≥ 16	≤ 4/8/≥ 16	≤ 4/8/≥ 16	≤ 4/8/≥ 16	≤ 4/8/≥ 16	≤ 4/8/≥ 16	≤ 4/8/≥ 16	≤ 4/8/≥ 16
Amoxicillin-clavulanate	-	≤ 8/16/≥ 32	≤ 8/16/≥ 32	≤ 8/16/≥ 32	≤ 8/16/≥ 32	≤ 8/16/≥ 32	≤ 8/16/≥ 32	≤ 8/16/≥ 32	≤ 8/16/≥ 32	≤ 8/16/≥ 32	≤ 8/16/≥ 32
Cefovecin	-	-	-	-	-	-	-	-	-	-	-
Chloramphenicol	≤ 8/16/≥ 32	≤ 8/16/≥ 32	≤ 8/16/≥ 32	≤ 8/16/≥ 32	≤ 8/16/≥ 32	≤ 8/16/≥ 32	≤ 8/16/≥ 32	≤ 8/16/≥ 32	≤ 8/16/≥ 32	≤ 8/16/≥ 32	≤ 8/16/≥ 32
Cefazolin	-	≤ 2/4/≥ 8	≤ 2/4/≥ 8	≤ 2/4/≥ 8	≤ 2/4/≥ 8	≤ 2/4/≥ 8	-	≤ 2/4/≥ 8	≤ 2/4/≥ 8	-	-
Imipenem	≤ 4/8/≥ 16	≤ 1/2/≥ 4	≤ 1/2/≥ 4	≤ 1/2/≥ 4	≤ 1/2/≥ 4	≤ 1/2/≥ 4	≤ 1/2/≥ 4	≤ 1/2/≥ 4	≤ 1/2/≥ 4	≤ 1/2/≥ 4	≤ 1/2/≥ 4
Marbofloxacin	-	-	-	-	-	-	-	-	-	-	-
Enrofloxacin	-	-	-	-	-	-	-	-	-	-	-
Amikacin	≤ 16/32/≥ 64	≤ 16/32/≥ 64	≤ 16/32/≥ 64	≤ 16/32/≥ 64	≤ 16/32/≥ 64	≤ 16/32/≥ 64	-	≤ 16/32/≥ 64	≤ 16/32/≥ 64	-	-
Cefpodoxime	-	≤ 2/4/≥ 8	≤ 2/4/≥ 8	≤ 2/4/≥ 8	≤ 2/4/≥ 8	≤ 2/4/≥ 8	≤ 2/4/≥ 8	≤ 2/4/≥ 8	≤ 2/4/≥ 8	≤ 2/4/≥ 8	≤ 2/4/≥ 8
Tetracycline	≤ 4/8/≥ 16	≤ 4/8/≥ 16	≤ 4/8/≥ 16	≤ 4/8/≥ 16	≤ 4/8/≥ 16	≤ 4/8/≥ 16	≤ 4/8/≥ 16	≤ 4/8/≥ 16	≤ 4/8/≥ 16	≤ 4/8/≥ 16	≤ 4/8/≥ 16
Cephalexin	-	≤ 2/4/≥ 8	≤ 2/4/≥ 8	≤ 2/4/≥ 8	≤ 2/4/≥ 8	≤ 2/4/≥ 8	≤ 2/4/≥ 8	≤ 2/4/≥ 8	≤ 2/4/≥ 8	≤ 2/4/≥ 8	≤ 2/4/≥ 8
Gentamicin	≤ 4/8/≥ 16	≤ 4/8/≥ 16	≤ 4/8/≥ 16	≤ 4/8/≥ 16	≤ 4/8/≥ 16	≤ 4/8/≥ 16	-	≤ 4/8/≥ 16	≤ 4/8/≥ 16	-	-

-

Supplemental Table 3: Potential pathogenic bacterial isolates from the semen of yearling bulls

Bacteria Name	Isolate ID	Number of Isolates	Media	Disease association in Cattle (reference)
Arthrobacter gandavensis	6502.d2_CB-C.sem<br>6503.d2_CB-C.sem<br>6504.d2_CB-C.sem<br>6530.d2_CB-F.sem<br>6540.d2_CB-B.sem<br>6552.d2_CB-E.sem<br>6570.d2_CB-E.sem<br>6573.d2_CB-C.sem<br>6577.d2_CB-E.sem<br>6588.d2_CB-E.sem<br>6593.d2_CB-A.sem<br>6617.d2_CB-A.sem<br>6703.d2_CB-A.sem<br>6740.d2_CB-C.sem<br>6743.d2_CB-A.sem<br>6749.d2_CB-D.sem<br>6752.d2_CB-B.sem	17 (Aerobic)	CB	Reproductive loss (Todhunter et al., 2013)<br>Mastitis (Santos et al., 2020)
Bacillus cereus	6460.d3_CB-C.sem<br>6540.d3_CB-B.sem<br>6540.d3_CB-C.sem<br>6546.d2_CB-E.sem<br>6577.d2_CB-H.sem<br>6641.d2_MRS-C.sem<br>6641.d3_MRS-B.sem<br>6546.d2_CB-B<br>6574.d2_CB-A<br>6577.d2_CB-A<br>6593.d3_CB-B<br>6709.d2_CB-A<br>6774.d2_CB-B	7 (Aerobic)<br>6 (Anaerobic)	CB (9)<br>MRS (2)	Abortion (Schuh and Weinstock, 1985; Alssahen et al., 2020)
Fusobacterium gastrosuis	6469.d2_CB-C	1 (Anaerobic)	CB	Footrot and lameness (Zhou et al., 2010)
Fusobacterium necrophorum	6460.d3_Bld-B<br>6469.d3_CB-A<br>6469.d3_CB-B<br>6540.d3_CB-A	6 (Anaerobic)	CB/Blood	Liver abscesses (Amachawadi and Nagaraja, 2016)

	6540.d3_CB-B<br>6540.d3_CB-C			
Histophilus somni	6503.d2_CB-E<br>6752.d3_CB-B	2 (Anaerobic)	CB	Bovine respiratory disease (Griffin et al., 2010)
Mannheimia varigena	6593.d3_CB-A.sem	1 (Aerobic)	CB	Bovine respiratory disease (Griffin et al., 2010)<br>Kidney infection (Komatsu et al., 2019)
Trueperella abortisuis	6546.d3_CB-A	1 (Anaerobic)	CB	Abortion (Alssahen et al., 2020)
Trueperella pyogenes	6641.d3_CB-C.sem	1 (Aerobic)	CB	Liver abscesses (Amachawadi and Nagaraja, 2016)
Bacillus licheniformis	6601.d2_CB-F<br>6617.d2_CB-F	2 (Anaerobic)	CB	Abortion (70)
Bacillus sonorensis	6574.d2_CB-C.sem<br>6601.d2_CB-E	1(Aerobic)<br>1(Anaerobic)	CB	Endometritis (71)
Enterococcus faecium	6502.d2_MRS-B.sem<br>6530.d2_MRS-B.sem<br>6552.d2_MRS-C.sem<br>6570.d2_MRS-C.sem<br>6641.d2_MRS-B.sem<br>6012.d2_CB-B<br>6347.d2_CB-A<br>6520.d2_CB-B<br>6546.d2_CB-A<br>6593.d2_CB-A<br>6601.d2_CB-A<br>6617.d2_CB-E<br>6641.d2_CB-C<br>6774.d2_CB-C	5 (Aerobic)<br>9 (Anaerobic)	MRS<br>CB	Mastitis (72)
Escherichia fergusonii	6502.d2_CB-D.sem<br>6530.d2_MRS-A.sem<br>6535.d2_CB-C.sem<br>6540.d2_CB-D.sem<br>6552.d2_CB-F.sem<br>6552.d2_MRS-B.sem<br>6593.d3_CB-D.sem<br>6641.d3_MRS-A.sem<br>6656.d2_CB-G.sem<br>6656.d2_MRS-A.sem<br>6709.d2_CB-B.sem<br>6740.d2_CB-B.sem<br>6749.d2_CB-A.sem<br>6774.d2_MRS-D.sem	16 (Aerobic)<br>17 (Anaerobic)	CB/Blood<br>MRS	Diarhhea (73)<br>Pneumonia (74)

	6865.d2_CB-D.sem<br>6865.d2_MRS-B.sem<br>6347.d2_CB-B<br>6520.d2_CB-D<br>6530.d2_Bld-B<br>6540.d2_Bld-A<br>6540.d2_Bld-D<br>6577.d2_CB-D<br>6577.d2_CB-E<br>6593.d3_CB-C<br>6593.d3_CB-D<br>6601.d2_CB-B<br>6641.d2_CB-E<br>6641.d3_CB-B<br>6641.d3_CB-C<br>6749.d2_CB-D<br>6787.d3_CB-C<br>6865.d2_CB-A<br>6641.d3_CB-C			
Lactococcus formosensis	6601.d2_MRS-D.sem	1 (Aerobic)	MRS	Mastitis (75)
Salmonella enterica	6740.d2_MRS-A.sem	1 (Aerobic)	MRS	Enteric, septicemic, and reproductive diseases (76)
Serratia liquefaciens	6504.d2_CB-B.sem<br>6520.d2_MRS-C.sem<br>6527.d2_CB-B.sem<br>6527.d2_CB-E.sem<br>6535.d2_CB-E.sem	5 (Aerobic)	CB<br>MRS	Mastitis (77)
Serratia quinivorans	6520.d2_CB-E.sem<br>6546.d2_CB-C.sem<br>6570.d2_CB-D.sem<br>6703.d2_CB-B.sem	4 (Aerobic)	CB	Mastitis (77)
Shigella flexneri	6570.d2_CB-B.sem<br>6513.d2_CB-A<br>6530.d2_Bld-C<br>6574.d2_CB-D	1 (Aerobic)<br>3 (Anaerobic)	CB/Blood	Mastitis (78)
Staphylococcus chromogenes	6460.d3_CB-D.sem<br>6460.d3_CB-E.sem<br>6469.d3_CB-B.sem<br>6530.d2_CB-D.sem<br>6540.d3_CB-A.sem<br>6546.d3_CB-B.sem	21 (Aerobic)<br>5 (Anaerobic)	CB/Blood<br>MRS	Mastitis (79)

	6570.d2_CB-F.sem<br>6573.d2_CB-B.sem<br>6577.d2_CB-A.sem<br>6588.d2_CB-C.sem<br>6593.d2_CB-B.sem<br>6593.d2_CB-C.sem<br>6593.d3_CB-C.sem<br>6641.d3_CB-A.sem<br>6656.d2_CB-D.sem<br>6709.d2_CB-D.sem<br>6752.d3_CB-C.sem<br>6752.d3_MRS-A.sem<br>6760.d3_CB-C.sem<br>6760.d3_MRS-A.sem<br>6787.d3_CB-C.sem<br>6012.d2_CB-C<br>6460.d3_Bld-A<br>6546.d3_CB-B<br>6752.d3_CB-D<br>6760.d2_CB-A			
Staphylococcus simulans	6502.d2_CB-F.sem<br>6504.d2_CB-A.sem<br>6513.d2_CB-D.sem<br>6546.d2_CB-B.sem<br>6552.d2_CB-D.sem<br>6601.d2_CB-B.sem<br>6641.d2_CB-B.sem<br>6546.d2_CB-D<br>6574.d2_CB-B<br>6749.d2_CB-C	7 (Aerobic)<br>3 (Anaerobic)	CB	Mastitis (79)
Staphylococcus haemolyticus	6513.d3_CB-B.sem	1 (Aerobic)	CB	Mastitis (80)
Streptococcus uberis	6469.d3_MRS-B.sem<br>6593.d3_MRS-A.sem<br>6460.d2_CB-A<br>6460.d3_Bld-D<br>6530.d2_Bld-A	2 (Aerobic)<br>3 (Anaerobic)	MRS<br>CB/Blood	Mastitis (81)
